# Causal Effects of Gut Microbiota on Sleep-Related Phenotypes: A Two-Sample Mendelian Randomization Study

**DOI:** 10.3390/clockssleep5030037

**Published:** 2023-09-12

**Authors:** Min Yue, Chuandi Jin, Xin Jiang, Xinxin Xue, Nan Wu, Ziyun Li, Lei Zhang

**Affiliations:** 1 Department of Biostatistics, School of Public Health, Cheeloo College of Medicine, Shandong University, Jinan 250012, China; 2 Microbiome-X, National Institute of Health Data Science of China & Institute for Medical Dataology, Cheeloo College of Medicine, Shandong University, Jinan 250012, China; 3 State Key Laboratory of Microbial Technology, Shandong University, Qingdao 266237, China

**Keywords:** gut microbiota, Mendelian randomization study, chronotype, sleep quality, abnormal sleep patterns

## Abstract

Increasing evidence suggests a correlation between changes in the composition of gut microbiota and sleep-related phenotypes. However, it remains uncertain whether these associations indicate a causal relationship. The genome-wide association study summary statistics data of gut microbiota (*n* = 18,340) was downloaded from the MiBioGen consortium and the data of sleep-related phenotypes were derived from the UK Biobank, the Medical Research Council-Integrative Epidemiology Unit, Jones SE, the FinnGen consortium. To test and estimate the causal effect of gut microbiota on sleep traits, a two-sample Mendelian randomization (MR) approach using multiple methods was conducted. A series of sensitive analyses, such as horizontal pleiotropy analysis, heterogeneity test, MR Steiger directionality test and “leave-one-out” analysis as well as reverse MR analysis, were conducted to assess the robustness of MR results. The genus *Anaerofilum* has a negative causal effect on getting up in the morning (odd ratio = 0.977, 95% confidence interval: 0.965–0.988, *p* = 7.28 × 10^−5^). A higher abundance of order Enterobacteriales and family Enterobacteriaceae contributed to becoming an “evening person”. Six and two taxa were causally associated with longer and shorter sleep duration, respectively. Specifically, two SCFA-produced genera including *Lachnospiraceae UCG004* (odd ratio = 1.029, 95% confidence interval = 1.012–1.046, *p* = 6.11 × 10^−4^) and *Odoribacter* contribute to extending sleep duration. Two obesity-related genera such as *Ruminococcus torques* (odd ratio = 1.024, 95% confidence interval: 1.011–1.036, *p* = 1.74 × 10^−4^) and *Senegalimassilia* were found to be increased and decreased risk of snoring, respectively. In addition, we found two risk taxa of insomnia such as the order Selenomonadales and one of its classes called Negativicutes. All of the sensitive analysis and reverse MR analysis results indicated that our MR results were robust. Our study revealed the causal effect of gut microbiota on sleep and identified causal risk and protective taxa for chronotype, sleep duration, snoring and insomnia, which has the potential to provide new perspectives for future mechanistic and clinical investigations of microbiota-mediated sleep abnormal patterns and provide clues for developing potential microbiota-based intervention strategies for sleep-related conditions.

## 1. Introduction

Sleep is an essential physiological activity that takes up one-third of human life and is crucial to the proper functioning of the body [1]. However, despite the widespread recognition of the importance of sleep, sleep disturbances are becoming increasingly prevalent in contemporary society and can lead to a multitude of severe health conditions [2,3]. Individuals who suffer from harmful sleep patterns and inadequate sleep duration often experience a higher burden of psychological and physical health issues, leading to considerable distress and suffering [4,5,6]. Numerous prior studies have established strong associations between disrupted sleep, including sleep fragmentation, sleep deprivation, obstructive sleep apnea, insomnia, and a range of health conditions, including cardiovascular diseases [7], obesity [8,9], diabetes [10], mental illness [11] and even mortality [12,13].

The gut microbiota, which exists within the digestive system, holds the potential to significantly impact human life as an environmental factor. A plethora of observational studies have indicated that the gut microbiota could play a role in either increasing or reducing the risk of different diseases, such as insomnia [14], and is intimately linked to the regulation of circadian rhythm [15]. However, it is important to note that these observational studies face challenges in effectively addressing confounding factors, such as age, environment, dietary patterns, and lifestyle [16] which can significantly impact the findings. These limitations greatly restrict the ability to establish definitive cause-and-effect relationships between gut microbiota and sleep characteristics.

In contrast to observational studies, the utilization of Mendelian randomization (MR) analysis capitalizes on the inherent characteristics of common genetic variations relating to modifiable environmental exposures that are of interest. This approach has gained significant popularity as a means to investigate the possible causal connections between environmental exposures and diseases [17,18,19]. Two-sample MR analysis incorporates data from separate Genome-Wide Association Studies (GWASs) on single-nucleotide polymorphisms (SNPs) related to the exposure and the outcome of interest. This approach allows for the calculation of a single causal estimate. As the number of GWASs focusing on the gut microbiota and various diseases continues to increase [20,21], there is now a greater abundance of large-scale summary statistics data available. This abundance has greatly enhanced the statistical power of two-sample MR analysis. In this research, we examined the causal relationship between the gut microbiota and sleep-related traits and diseases by conducting a comprehensive two-sample MR analysis of three kinds of sleep-related phenotypes derived from the IEU Open GWAS project, including chronotype (chronotype and getting up in morning), sleep quality (sleep duration, snoring and nap during day), and abnormal sleep patterns(insomnia, narcolepsy, disorder of the sleep-wake schedule, sleep disorders and sleep apnoea). Several taxa that play a causal role in the chronotype, sleep duration and the risk of both insomnia and snoring were further identified which contributes to exploring the mechanism.

## 2. Results

**Figure 1:**
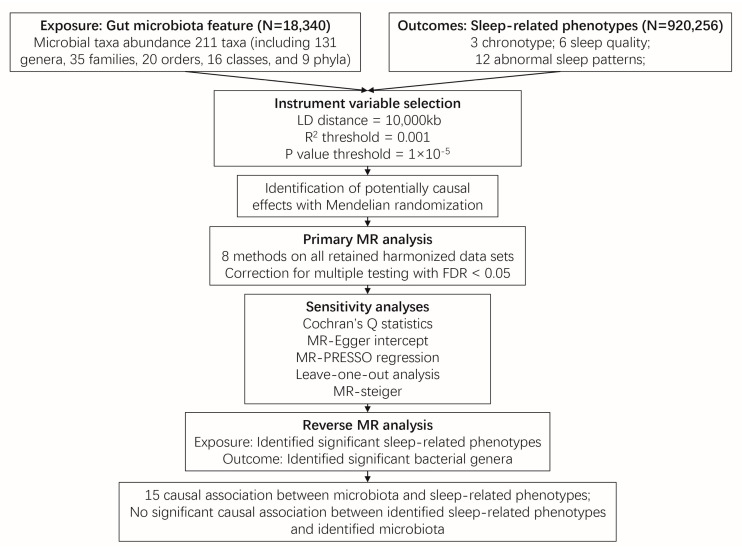
Overview of the two sample Mendelian randomization framework used to investigate the causal effect of gut microbiota on sleep-related phenotypes. Abbreviation: MR, Mendelian randomization.

Figure 1 illustrates the conceptual framework of this MR analysis. The main objective of this MR analysis was to examine the hypothesis that the gut microbiome has a causal impact on chronotype, sleep quality, and abnormal sleep patterns. The analysis aimed to provide estimates for each association between the exposure (gut microbiome) and the outcomes (chronotype, sleep quality, and abnormal sleep patterns). Based on the 211 bacterial taxa GWAS summary data in the MiBioGen consortium, 15 taxa without exact annotation (unknown family or genus) and 1 taxon with no IVs were excluded, resulting in a total of 195 taxa being retained for the following research. In which, a weak IV with an F value less than 10 was removed. Finally, 125, 224, 280, 434, and 1495 SNPs were identified as IVs for 9 phylum, 16 class, 20 order, 31 family, and 119 genera, respectively. Detailed information about the selected IVs is provided in Table S1. The harmonized dataset of the associations between genetic variant and exposure, and between genetic variant and outcome is presented in Table S2.

**Figure 2:**
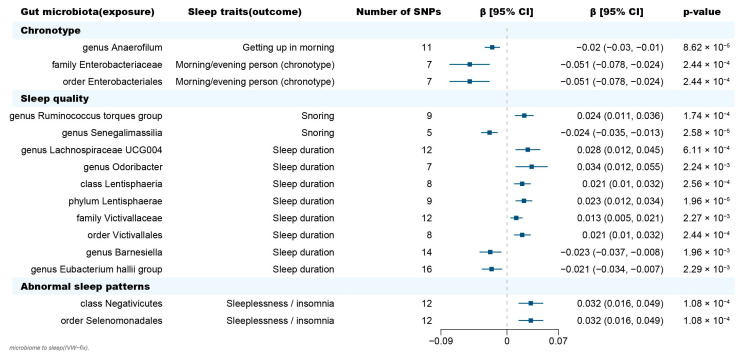
Forest plot of causal effects of gut microbiota on sleep-related phenotypes. Abbreviations: SNP, single nucleotide polymorphism; β, the ratio estimates; CI, confidence interval; IVW, inverse-variance weighted method.

The Inverse-Variance Weighted (IVW) method, being a conventional MR approach, is known to exhibit slightly higher statistical power compared to other methods under specific conditions [22]. We mainly considered the causal estimate between 195 bacterial taxa and sleep traits (chronotype, sleep quality and abnormal sleep patterns), with the results derived from the other seven MR methods serving as complements. After Benjamini-Hochberg correction for multiple testing, 26 significant causal relationships with FDR < 0.05 were identified (Tables S3–S5). After removing the MR results with heterogeneity (Cochran’s Q statistics *p* < 0.05), horizontal pleiotropy (MR-Egger intercept *p* < 0.05, the MR-PRESSO global test *p* < 0.05) that was driven by a single SNP (leave-one-out analysis), 15 causal relationships were retained (Figure 2, Table 1, Figure S1 and Table S6). The MR Steiger directionality test indicated that in all these 15 causal relationships, taxa served as exposure and the sleep phenotypes served as outcome (*p* < 0.05) (Table 1). Furthermore, the causal estimates in these 15 causal relationships were consistent with those in the other 7 MR methods (Figure S2 and Table S7).

### 2.1. Causal Effect of Gut Microbiota on Chronotype

**Figure 3:**
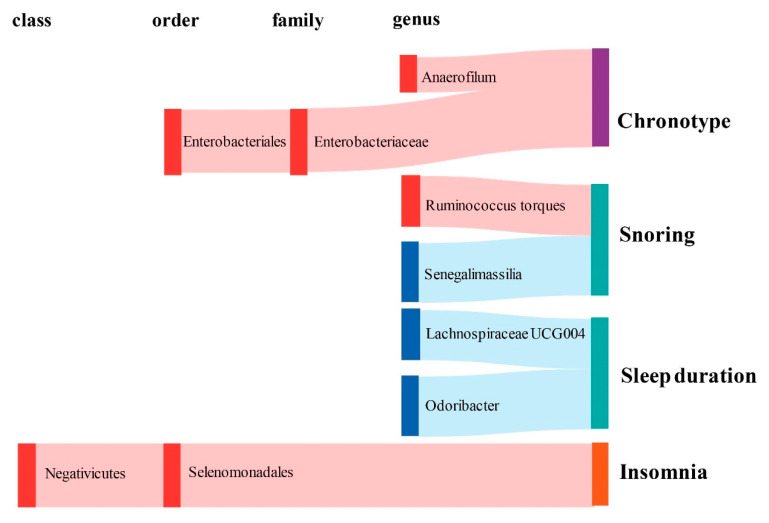
Graphical summary of the gut microbiota causally associated with sleep phenotypes and their subordination at different taxonomic levels. The gut microbiota is arranged at the taxonomic levels of class, order, family, and genus. In addition, red represents the β of the causal association is greater than 0, while blue represents the β of the causal association is less than 0. Sleep phenotypes are classified into three colors: purple represents circadian types, green represents sleep quality (including sleep duration and snoring), and orange represents abnormal sleep patterns.

In terms of chronotype, the gut microbiota has causal effects on the uneasiness of getting up in the morning and becoming an “evening person” (Figure 3). We found that the genus *Anaerofilum* has a negative effect on getting up in the morning (odd ratio = 0.98, 95% confidence interval = 0.97–0.99, *p* = 8.62 × 10^−5^, IVW). Compared to “morning person”, individuals with higher abundance of order Enterobacteriales and family Enterobacteriaceae are more likely to be an “evening person”, based on its causal effect on morning/evening person (OR = 0.95, 95% CI = 0.925–0.977, *p* = 2.44 × 10^−4^; OR = 0.95, 95% CI = 0.925–0.977, *p* = 2.44 × 10^−4^, IVW).

### 2.2. Causal Effect of Gut Microbiota on Sleep Quality

Considering sleep quality, gut microbiota not only has a causal effect on sleep duration but also influences the snoring risk (Figure 3). A total of 6 bacteria taxa for extending sleep duration were found in this present study including the genus *Lachnospiraceae UCG004* (OR = 1.029, 95% CI = 1.012–1.046, *p* = 6.11 × 10^−4^, IVW), the genus *Odoribacter* (OR = 1.034, 95% CI = 1.012–1.057, *p* = 2.24 × 10^−3^, IVW), the family Victivallaceae (OR = 1.013, 95% CI = 1.005–1.021, *p* = 2.27 × 10^−3^, IVW), the order Victivallales (OR = 1.021, 95% CI = 1.01–1.032, *p* = 2.56 × 10^−4^, IVW), the phylum Lentisphaerae (OR = 1.023, 95% CI = 1.013–1.034, *p* = 1.96 × 10^−5^, IVW) and the class Lentisphmulaeria (OR = 1.021, 95% CI = 1.010–1.032, *p* = 2.56 × 10^−4^, IVW). While, a higher abundance of both genus Barnesiella (OR = 0.977, 95% CI = 0.963–0.992, *p* = 1.96 × 10^−3^, IVW) and Eubacterium hallii (OR = 0.979, 95% CI = 0.966–0.993, *p* = 2.29 × 10^−3^, IVW) are causally associated with shorter sleep duration. The genus *Senegalimassilia* (OR = 0.976, 95% CI = 0.966–0.987, *p* = 2.58 × 10^−5^, IVW) and genus *R. torques* (OR = 1.024, 95% CI = 1.011–1.036, *p* = 1.74 × 10^−4^, IVW) were causally associated with decreased and increased snoring risk, respectively.

### 2.3. Causal Effect of Gut Microbiota on Abnormal Sleep Patterns

Considering abnormal sleep patterns, two taxa were found to be associated with increased insomnia risk in our results (Figure 3). Both the order Selenomonadales and class Negativicutes were risk taxa for insomnia (OR = 1.033, 95% CI = 1.016–1.05, *p* = 1.08 × 10^−4^; OR = 1.033, 95% CI = 1.016–1.05, *p* = 1.08 × 10^−4^, IVW).

### 2.4. Bidirectional Causal Effects between Gut Microbiota and Sleep Phenotypes

Based on the results from the reverse MR analysis, no significant causal effect of sleep phenotypes on the abundance of gut microbiota was observed according to the IVW results (Tables S8 and S9). Additionally, all these results were robust and not biased by heterogeneity (Cochran’s Q statistics *p* > 0.05) and horizontal pleiotropy (MR-Egger intercept *p* > 0.05, the MR-PRESSO global test *p* > 0.05) in Table S10. No causal relationship was derived by a single SNP (Table S11).

## 3. Discussion

Observational studies have revealed a close correlation between gut microbiota and sleep habits and sleep-related disorders [23,24,25,26,27,28,29,30]. The presence of healthy gut microbiota is crucial for the proper regulation and preservation of normal sleep patterns. Meanwhile, previous research has shown that disrupted sleep patterns and inadequate sleep duration can influence the composition, diversity, and functioning of the gut microbiota through the brain-gut-microbiota axis (BGMA) [31]. However, the relationship between gut microbiota and sleep-related phenotypes remains primarily unrecognized or uncertain in terms of cause and effect. MR has been extensively utilized to investigate the causal relationship between sleep traits and various diseases including cognitive disorders [32], autoimmune diseases [33,34], and psychiatric disorders [32]. By using the two-sample MR method, we demonstrated the causal effect of gut microbiota on chronotype, sleep quality and abnormal sleep patterns, and identified causal taxa that influence get up habit, sleep duration, snoring as well as risk taxa for insomnia in this present study.

Sleep duration plays a critical role in determining the quality of sleep and has been associated with various aspects of cognitive and neurobehavioral functioning [35,36,37]. Moreover, insufficient sleep duration has been correlated with the increased risk of developing various health conditions, such as cancer [38], type II diabetes [39,40], and Alzheimer’s disease [41]. In this present study, six bacteria taxa, especially two short-chain fatty acids (SCFAs e.g., propionate, acetate, butyrate)-produced genera including *Lachnospiraceae UCG004* and *Odoribacter* were found to contribute to extending sleep duration. As a major microbiota’s fermentation product of fiber fermentation in the gut, SCFAs could affect sleep via gut–brain communications. This phenomenon is attributed to the release of gastrointestinal hormones like glucagon-like peptide 1 and peptide YY, as well as neurotransmitters gamma-aminobutyric acid (GABA) and serotonin [42]. Among which, GABA, the main inhibitory neurotransmitter in the central nervous system, plays a significant role in promoting sleep as it inhibits various arousal systems responsible for wakefulness [43]; the serotonergic system was found to promote sleep by generating homeostatic sleep pressure in both zebrafish and rodents during wakefulness. Furthermore, a higher concentration of propionate in total fecal samples has been found to be strongly correlated with longer uninterrupted infant sleep. Szentirmai et al. found that when tributyrin and butyrate, which are SCFAs, were given orally or intraportally, they effectively enhanced non-rapid-eye movement sleep (NREMS) in rats and mice by acting on the liver and/or portal vein [44]. Hence, we speculated that SCFAs produced by increased *Lachnospiraceae UCG004* and *Odoribacter* may improve sleep duration through modulating GABA and serotonin activity in the brain via gut–brain communications. Besides, the family Victivallaceae and the order Victivallales were detected to be causally correlated with shorter sleep duration. A lower abundance of the family Victivallaceae has been found in people with autoimmune-based thyroid diseases [45], while lack of sleep can result in an imbalance of oxidative processes in the thyroid, leading to thyroid damage in the long run [46]. Whether the family Victivallaceae serve as a risk factor for thyroid function impairment induced by sleep deprivation needs further research.

Insomnia is defined as a condition that involves difficulties in inducing or sustaining sleep and/or non-restorative sleep, along with a decrease in daytime functioning, which lasts for at least four weeks [47]. Patients with insomnia often exhibit various alterations in the composition and functioning of their gut microbiota [15]. Melatonin (N-acetyl-5-methoxytryptamine), the predominant hormone released by the pineal gland, is commonly used to improve sleep in patients with insomnia [48] which can also relieve sleep deprivation-induced gut microbiota disorders. Reduced abundance of the order Selenomonadales was observed in sucking piglets treated with melatonin [49]. In our study, a higher abundance of order Selenomonadales and one of its classes, Negativicutes, are closely associated with increased insomnia risk. These findings suggest that the Selenomonadales order and Negativicutes class may significantly contribute to the effectiveness of melatonin in alleviating insomnia symptoms, potentially by enhancing the development of intestinal neural pathways and promoting intestinal barrier function [49].

In addition to its impact on sleep duration and insomnia, gut microbiota may also play a role in an individual’s chronotype. Chronotype refers to an individual’s behavioral tendencies based on their circadian rhythm [50], subdivided into evening person and morning person, commonly referred to as “night owls” and “early larks” [51]. Due to the internal biological clock being shifted, the evening person finds it easier to stay awake at night and has more difficulty waking up early. This chronotype is more prone to present an increased body weight and body mass index (BMI) compared to the morning chronotypes [52,53]. In this study, we identified three obesity-related bacteria such as the genus *Anaerofilum*, the order Enterobacteriales, and one of its family, Enterobacteriaceae contribute to becoming an evening person. Specifically, the genus *Anaerofilum* belongs to the Firmicutes, and is known to be one of the “obesogenic microbiota” [54]. In a previous MR study, the family Enterobacteriaceae have been demonstrated to be causally associated with insulin resistance and obesity [55]. Furthermore, Wang et al. reported that gut microbiota could regulate the circadian clock gene NFIL3 of the intestinal epithelium, thereby altering people’s BMI [56,57]. These findings suggested that these three bacteria may impact the circadian clock genes of the intestinal epithelium, leading people to be more prone to evening-type chronotypes and ultimately to metabolic disorders such as obesity.

Obesity, as a health condition, is known to increase the risk of sleep-disordered breathing, such as snoring [58,59,60]. This research found a causal link between obesity-related genera and snoring risk. Specifically, individuals with a higher abundance of the genus *R. torques* are found to be prone to snoring, while the genus *Senegalimassilia* serves as a beneficial taxon against snoring. The genus *R. torques* and Ruminococcus gnavus have been previously documented to be highly enriched in obese individuals [61] and be correlated with metabolic disorders [62] and inflammatory bowel diseases [63]. On the other hand, a reduction in the abundance of *Senegalimassilia* anaerobia (a species within the *Senegalimassilia* genus) has been noticed in the fecal samples of overweight children [64]. These findings further support the association between gut microbiota, obesity, and sleep-related conditions.

This study possesses several notable strengths. Firstly, the implementation of MR analysis allowed for the assessment of a causal relationship between gut microbiota and sleep-related phenotypes, effectively removing the interference of confounding factors and reversing causation. Another strength of this research is the utilization of genetic variants of gut microbiota-derived from the largest GWAS meta-analysis available. This approach ensured the robustness and strength of the instruments used in the MR analysis. Moreover, the sensitivity analysis and reverse MR analysis results showed our results are statistically robust. To minimize bias, a two-sample MR design was employed, utilizing non-overlapping summary-level data for the exposure and outcome variables.

However, it is important to acknowledge the limitations of this study. Firstly, the GWAS summary data used predominantly consisted of patients of European descent, which may restrict the generalizability of our findings. Secondly, the study was restricted to the genus level of taxonomic classification in the exposure dataset, which made it difficult to explore the causal relationship between gut microbiota at the species level and sleep-related phenotypes. Lastly, to perform sensitivity analysis and identify horizontal pleiotropy, a larger number of IVs would be required. Consequently, the SNPs used in the analysis did not meet the conventional GWAS significance threshold (*p* < 5 × 10^−8^). For this, a false discovery rate (FDR) correction was implemented to minimize the potential for false positives.

## 4. Materials and Methods

### 4.1. Exposure Data

The GWAS summary statistics data relating to the abundance of human gut microbiome was derived from the international multi-ethnic consortium MiBioGen’s GWAS dataset [21]. This large-scale GWAS project involved 18,340 participants from 24 cohorts in various countries such as the USA, Canada, Germany, Denmark, Belgium, Sweden, Finland, the UK, The Netherlands, Israel, and South Korea. The project coordinated sequencing profiles of the 16S ribosomal RNA gene, as well as genotyping data, to examine the relationship between autosomal human genetic variants and the gut microbiome. A total of 211 taxa were analyzed, comprising 131 genera, 35 families, 20 orders, 16 classes, and 9 phyla.

### 4.2. Outcome Data

We downloaded the GWAS summary statistics data of three sleep-related phenotypes, including chronotype (chronotype and getting up in the morning), sleep quality (sleep duration, snoring and nap during the day), and abnormal sleep patterns (insomnia, narcolepsy, disorder of the sleep-wake schedule, sleep disorders and sleep apnoea) from the IEU Open GWAS project, UK Biobank [65], the Medical Research Council-Integrative Epidemiology Unit (MRC-IEU) [66], Jones SE [67], and the FinnGen consortium (https://www.finngen.fi/en/access_results, accessed on 17 June 2022). Detailed information on sleep phenotypes [67,68,69,70,71] is provided in Table 2 and Table S12.

### 4.3. Instrumental Variable (IV) Selection

To select instrumental variables (IVs) for our analysis, we used the following screening process: (1) Single-nucleotide polymorphisms (SNPs) that are significantly associated with each sleep phenotype, using a threshold of locus-wide significance (*p* < 1.0 × 10^−5^), were identified; (2) Considering the presence of strong linkage disequilibrium (LD) might result in biased results, the clumping process (R^2^ < 0.01 and clumping distance = 10,000 kb) was conducted; (3) Remove SNPs with minor allele frequency (MAF) ≤ 0.01; and (4) In cases where palindromic SNPs were present, the alleles on the forward strand were determined by utilizing the information on allele frequencies; (5) the F-statistic was used as a measure to evaluate the effectiveness of IVs [72]. IVs with an F-value less than 10 were considered weak instruments and excluded [72].

### 4.4. MR Analysis

In this present study, we used Cochran’s Q statistics to quantify the heterogeneity of IVs. In the absence of heterogeneity (*p* > 0.05), the inverse variance weighted fixed effects (IVW-fixed) method was employed to assess the presence of a causal link between gut microbiota and sleep phenotypes. Additionally, we used multiple methods including maximum likelihood, MR-Egger regression, weighted median, weighted model, Mendelian Randomization Pleiotropy RESidual Sum and Outlier (MR-PRESSO), simple median and simple mode method to complement our MR analysis on the association between gut microbiota and sleep phenotypes. By employing a meta-analysis technique and incorporating Wald estimates for each single nucleotide polymorphism (SNP), the IVW method derived a comprehensive estimate of the impact of gut microbiota on sleep. If horizontal pleiotropy, a potential confounding factor, was absent, the results obtained via the IVW method would be free from bias [73]. When both heterogeneity and horizontal pleiotropy are not present, the maximum likelihood method and the IVW method are similar [74]. The MR-Egger regression method incorporates the assumption of instrument strength independent of direct effect (InSIDE), allowing for the examination of pleiotropy by analyzing the intercept term. Specifically, if the intercept term is estimated to be equal to zero, it suggests the absence of horizontal pleiotropy. In this case, the results obtained from the MR-Egger regression align with those of the IVW method, further enhancing the credibility and consistency of the findings [75]. The weighted median method is valuable in estimating the causal association accurately, even in scenarios where up to half of the instrumental variables (IVs) used in the analysis are invalid [76]. When the InSIDE hypothesis is not upheld, it has been observed that the weighted model estimate outperforms MR-Egger regression in terms of enhanced power to detect a causal effect, reduced bias, and lower rates of type I error [76]. The MR-PRESSO analysis identifies and aims to minimize the impact of horizontal pleiotropy by eliminating notable outliers. However, it is essential to ensure that a minimum of 50% of the genetic variants used are reliable instruments and that the InSIDE assumptions are met in order to effectively utilize the MR-PRESSO analysis [77].

### 4.5. Sensitivity Analyses

The MR-PRESSO global test was employed to identify any potential pleiotropic effects. Additionally, the MR-Egger intercept test was utilized to evaluate the presence of horizontal pleiotropy in the MR analysis. To assess if the causal estimate was influenced by a single SNP, a “leave-one-out” analysis was conducted by systematically excluding each instrumental SNP one at a time. To investigate the directional causality of the exposure on the outcome, the MR Steiger directionality test was performed [78].

### 4.6. Reverse MR Analysis

To evaluate the potential causal relationship between sleep and gut microbiota, a reverse MR analysis was also conducted. This involved using the identified causal sleep phenotype as the exposure and investigating its impact on the gut microbiota as the outcome. The methods and settings employed in the reverse MR analysis were consistent with those used in the forward MR analysis.

### 4.7. Statistical Analyses

All statistical analyses were conducted using R version 4.0. IV selection, MR analyses, sensitive analysis and reverse MR analysis were performed using the TwosampleMR (version 0.5.6) [78] and MR-PRESSO (version 1.0) R packages [77].

## 5. Conclusions

Taken together, this study provides evidence supporting a cause-and-effect relationship between the gut microbiota and sleep-related phenotypes. The gut microbiota may affect sleep duration through the production of SCFAs that affect neurotransmitters such as GABA and serotonin. It may also be involved in thyroid dysfunction caused by inadequate sleep duration. In addition, the gut microbiota may also trigger obesity by regulating a person’s chronotype, and at the same time, it may affect the risk of snoring by regulating obesity. Based on our findings, we provide support for the impact of gut microbiota on sleep-related phenotypes and further provide clues for developing microbiota-based intervention strategies for sleep-related habits and disorders.

## Figures and Tables

**Table 1:** The heterogeneity, horizontal pleiotropy and MR Steiger results of the association between gut microbiota and sleep-related phenotypes.

Exposure	Outcome	Exposure ID	Outcome ID	Q	Q df	Q Pval	I^2^	Sample Size	Intercepts [95% CI]	Pval	Global Test RSSobs	Global Test Pvalue	Distortion Test Pvalue	Distortion Coefficient	Correct Causal Direction	Steiger Test
class Negativicutes id.2164	Sleeplessness/insomnia || id:ukb-b-3957	ebi-a-GCST90016922	ukb-b-3957	8.09	11.0	0.71	−0.36	14,306	−0.001 (−0.004, 0.003)	0.717	9.73	0.768	NA	NA	TRUE	3.77 × 10^−56^
order Selenomonadales id.2165	Sleeplessness/insomnia || id:ukb-b-3957	ebi-a-GCST90017107	ukb-b-3957	8.09	11.0	0.71	−0.36	14,306	−0.001 (−0.004, 0.003)	0.717	9.73	0.768	NA	NA	TRUE	3.77 × 10^−56^
class Lentisphaeria id.2250	Sleep duration || id:ukb-b-4424	ebi-a-GCST90016918	ukb-b-4424	10.40	7.0	0.17	0.33	14,306	0.003 (−0.004, 0.01)	0.368	13.60	0.273	NA	NA	TRUE	4.47 × 10^−42^
family Victivallaceae id.2255	Sleep duration || id:ukb-b-4424	ebi-a-GCST90016958	ukb-b-4424	15.80	11.0	0.15	0.30	14,306	0.002 (−0.004, 0.009)	0.524	18.90	0.212	NA	NA	TRUE	4.91 × 10^−62^
genus Barnesiella id.944	Sleep duration || id:ukb-b-4424	ebi-a-GCST90016969	ukb-b-4424	11.10	13.0	0.60	−0.17	14,306	−0.003 (−0.007, 0.001)	0.155	12.90	0.605	NA	NA	TRUE	2.55 × 10^−73^
genus Eubacterium hallii group id.11338	Sleep duration || id:ukb-b-4424	ebi-a-GCST90017000	ukb-b-4424	11.80	15.0	0.70	−0.27	14,306	0.001 (−0.001, 0.003)	0.344	13.20	0.738	NA	NA	TRUE	1.30 × 10^−67^
genus *Lachnospiraceae UCG004* id.11324	Sleep duration || id:ukb-b-4424	ebi-a-GCST90017026	ukb-b-4424	15.40	11.0	0.17	0.28	14,306	0.004 (0, 0.008)	0.08	18.30	0.228	NA	NA	TRUE	1.27 × 10^−54^
genus *Odoribacter* id.952	Sleep duration || id:ukb-b-4424	ebi-a-GCST90017034	ukb-b-4424	4.57	6.0	0.60	−0.31	14,306	0.002 (−0.004, 0.007)	0.55	6.36	0.610	NA	NA	TRUE	1.53 × 10^−39^
order Victivallales id.2254	Sleep duration || id:ukb-b-4424	ebi-a-GCST90017109	ukb-b-4424	10.40	7.0	0.17	0.33	14,306	0.003 (−0.004, 0.01)	0.37	13.60	0.273	NA	NA	TRUE	4.47 × 10^−42^
phylum Lentisphaerae id.2238	Sleep duration || id:ukb-b-4424	ebi-a-GCST90017115	ukb-b-4424	11.90	8.0	0.15	0.33	14,306	0.003 (−0.004, 0.01)	0.44	15.10	0.293	NA	NA	TRUE	2.42 × 10^−46^
genus *Ruminococcus torques* group id.14377	Snoring || id:ukb-b-17400	ebi-a-GCST90017066	ukb-b-17400	12.40	8.0	0.13	0.36	14,306	0.002 (−0.001, 0.004)	0.25	15.30	0.210	NA	NA	TRUE	2.18 × 10^−54^
genus *Senegalimassilia* id.11160	Snoring || id:ukb-b-17400	ebi-a-GCST90017068	ukb-b-17400	4.70	4.0	0.32	0.15	14,306	0 (−0.004, 0.005)	0.89	7.18	0.465	NA	NA	TRUE	1.11 × 10^−32^
genus *Anaerofilum* id.2053	Getting up in morning || id:ukb-b-2772	ebi-a-GCST90016965	ukb-b-2772	13.90	1.0	0.18	0.28	14,306	−0.002 (−0.009, 0.005)	0.52	16.80	0.217	NA	NA	TRUE	5.87 × 10^−53^
family Enterobacteriaceae id.3469	Morning/evening person (chronotype) || id:ukb-b-4956	ebi-a-GCST90016936	ukb-b-4956	9.82	6.0	0.13	0.39	14,306	−0.008 (−0.023, 0.007)	0.29	13.40	0.163	NA	NA	TRUE	1.33 × 10^−44^
order Enterobacteriales id.3468	Morning/evening person (chronotype) || id:ukb-b-4956	ebi-a-GCST90017098	ukb-b-4956	9.82	6.0	0.13	0.39	14,306	−0.008 (−0.023, 0.007)	0.29	13.40	0.163	NA	NA	TRUE	1.33 × 10^−44^

Abbreviations: Q, Cochran’s Q; df, degree of freedom; Pval, *p* value; RSSobs, observed residual sum of squares; NA, not applicable.

**Table 2:** Overview of the data source of sleep-related phenotypes.

Phenotype	Consortium	Date	Sample Size (Case)	nSNPs	Population	Gender	GWAS_ID
Disorder of the sleep-wake schedule	FinnGen	2021	216,354 (190)	16,380,459	European	Mixed	finn-b-F5_SLEEPWAKE
Sleep apnoea	FinnGen	2021	217,965 (16,761)	16,380,465	European	Mixed	finn-b-G6_SLEEPAPNO
Sleep disorders	FinnGen	2021	216,454 (2628)	16,380,458	European	Mixed	finn-b-F5_SLEEP
Sleep disorders, other/unspecified	FinnGen	2021	216,496 (332)	16,380,458	European	Mixed	finn-b-F5_SLEEP_NOS
Other sleep disorders	FinnGen	2021	177,660 (1553)	16,380,337	European	Mixed	finn-b-G6_SLEEPDISOTH
Nonorganic sleeping disorders	FinnGen	2021	218,792 (2214)	16,380,466	European	Mixed	finn-b-KRA_PSY_SLEEP_NONORG
Sleep disorders (combined)	FinnGen	2021	216,700 (19,155)	16,380,458	European	Mixed	finn-b-SLEEP
Sleep duration	MRC-IEU	2018	460,099	9,851,867	European	Mixed	ukb-b-4424
Snoring	MRC-IEU	2018	430,438 (270,007)	9,851,867	European	Mixed	ukb-b-17400
Nap during day	MRC-IEU	2018	462,400	9,851,867	European	Mixed	ukb-b-4616
Sleeplessness/insomnia	MRC-IEU	2018	462,341	9,851,867	European	Mixed	ukb-b-3957
Diagnoses—main ICD10: G47.3 Sleep apnoea	MRC-IEU	2018	463,010 (2320)	9,851,867	European	Mixed	ukb-b-16781
Daytime dozing/sleeping (narcolepsy)	MRC-IEU	2018	460,913	9,851,867	European	Mixed	ukb-b-5776
Diagnoses—secondary ICD10: G47.3 Sleep apnoea	MRC-IEU	2018	463,010 (1385)	9,851,867	European	Mixed	ukb-b-7853
Non-cancer illness code, self-reported: sleep apnoea	MRC-IEU	2018	462,933 (1510)	9,851,867	European	Mixed	ukb-b-9155
Getting up in the morning	MRC-IEU	2018	461,658	9,851,867	European	Mixed	ukb-b-2772
Morning/evening person (chronotype)	MRC-IEU	2018	413,343	9,851,867	European	Mixed	ukb-b-4956
Sleep duration (over sleepers)	Jones SE	2016	91,306 (10,102)	16,563,303	European	Mixed	ebi-a-GCST006685
Sleep duration (under sleepers)	Jones SE	2016	110,188 (28,980)	16,561,726	European	Mixed	ebi-a-GCST006686
sleep duration	UK Biobank	2016	128,266	16,761,226	European	Mixed	ieu-a-1088
Chronotype	UK Biobank	2016	128,266	17,032,431	European	Mixed	ieu-a-1087

Abbreviations: nSNP, number of single nucleotide polymorphism; GWAS, genome-wide association study.

## Data Availability

The dataset of gut microbiota analyzed during the current study is available in the MiBioGen repository, https://mibiogen.gcc.rug.nl/, accessed on 6 April 2023. The summary datasets of sleep-related phenotypes are available in the IEU Open GWAS project, https://gwas.mrcieu.ac.uk/, accessed on 17 June 2022.

## Supplementary Materials

The following supporting information can be downloaded at: https://www.mdpi.com/article/10.3390/clockssleep5030037/s1, Figure S1: Leave-one-out plots for the causal association between gut microbiota and sleep-related phenotypes. Figure S2: Scatter plots for the causal association between gut microbiota and sleep-related phenotypes. Table S1: Instrumental variables used in MR analysis of the association between gut microbiota and sleep-related phenotypes. Table S2: The harmonized dataset of each exposure and outcome. Table S3: The potential relationships between gut microbiota and sleep-related phenotypes. Table S4: The heterogeneity, horizontal pleiotropy and MR steiger results of the potential causal association. Table S5: Leave-one-out analysis of the potential causal association between gut microbiota and sleep-related phenotypes. Table S6: Leave-one-out analysis of the causal association between gut microbiota and sleep-related phenotypes. Table S7: Full result of MR estimates for the association between gut microbiota and sleep-related phenotypes. Table S8: IVs used in the MR analysis of the association between sleep-related phenotypes and gut microbiota. Table S9: Result of MR estimates for the association between sleep-related phenotypes and gut microbiota. Table S10: The heterogeneity and horizontal pleiotropy of the association between sleep-related phenotypes and gut microbiota. Table S11: Leave-one-out analysis of the causal association between sleep-related phenotypes and gut microbiota. Table S12: Detail information of sleep traits.

## Abbreviations

BGMA: Brain-gut-microbiota axis, BMI: body mass index, CI: Confidence interval, FDR: False discovery rate, GABA: Gamma-aminobutyric acid, GWAS: Genome-Wide Association Study, InSIDE: Instrument strength independent of direct effect, IV: Instrumental variable, IVW: Inverse variance weighted, IVW-fixed: Inverse variance weighted fixed effects, LD: Linkage disequilibrium, MAF: Minor allele frequency, MR: Mendelian randomization, MRC-IEU: Medical Research Council-Integrative Epidemiology Unit, MR-PRESSO: Mendelian Randomization Pleiotropy RESidual Sum and Outlier, NREMS: Non-rapid-eye movement sleep, OR: Odd ratio, SCFA: Short-chain fatty acid, SNP: Single-nucleotide polymorphism.
