# Supplementary figures and images for: Causal Effects of Gut Microbiota on Sleep-Related Phenotypes: A Two-Sample Mendelian Randomization Study
Source: Clocks Sleep. 2023 Sep 12;5(3):566–80. doi: 10.3390/clockssleep5030037 (PMC10527580; doi:10.3390/clockssleep5030037)

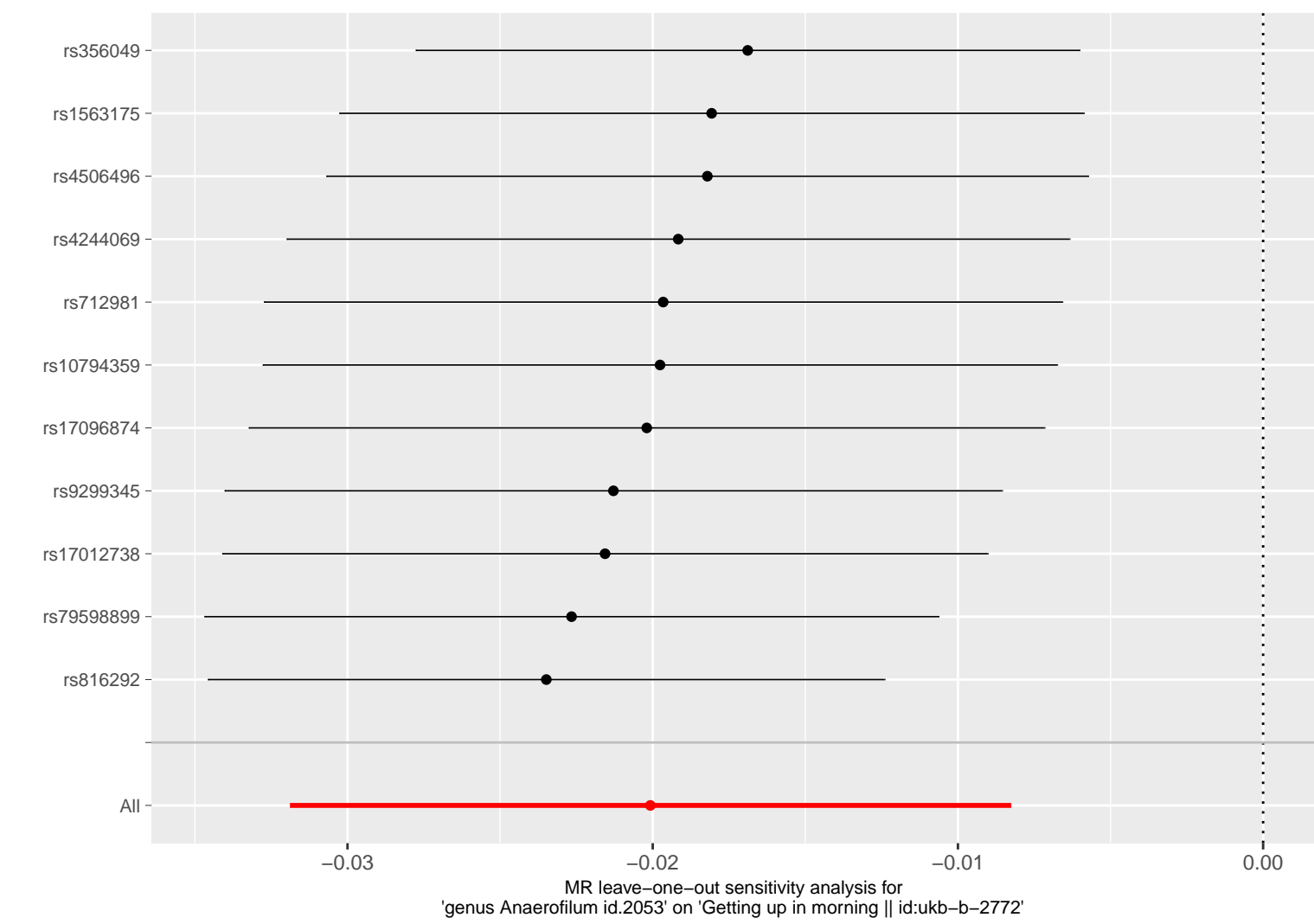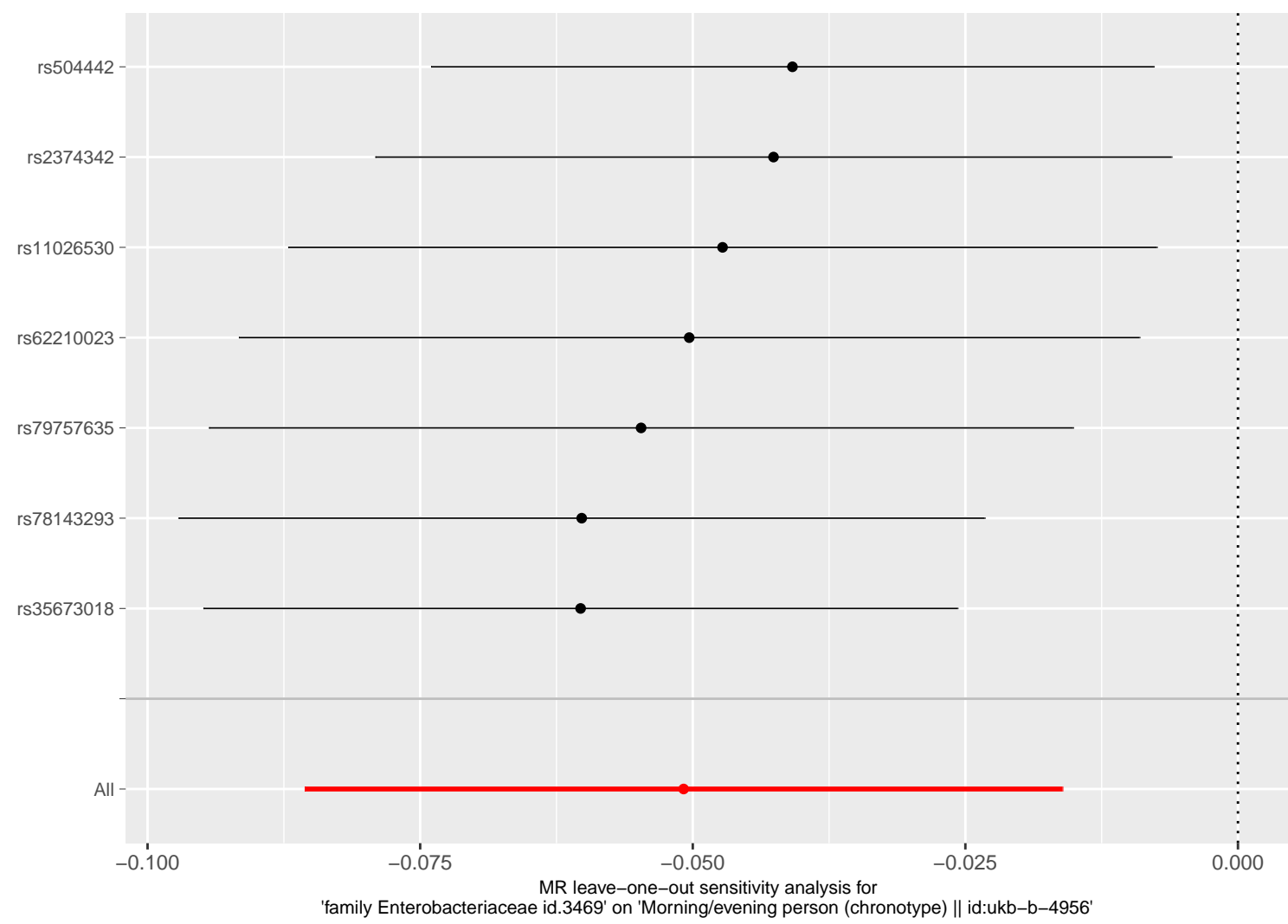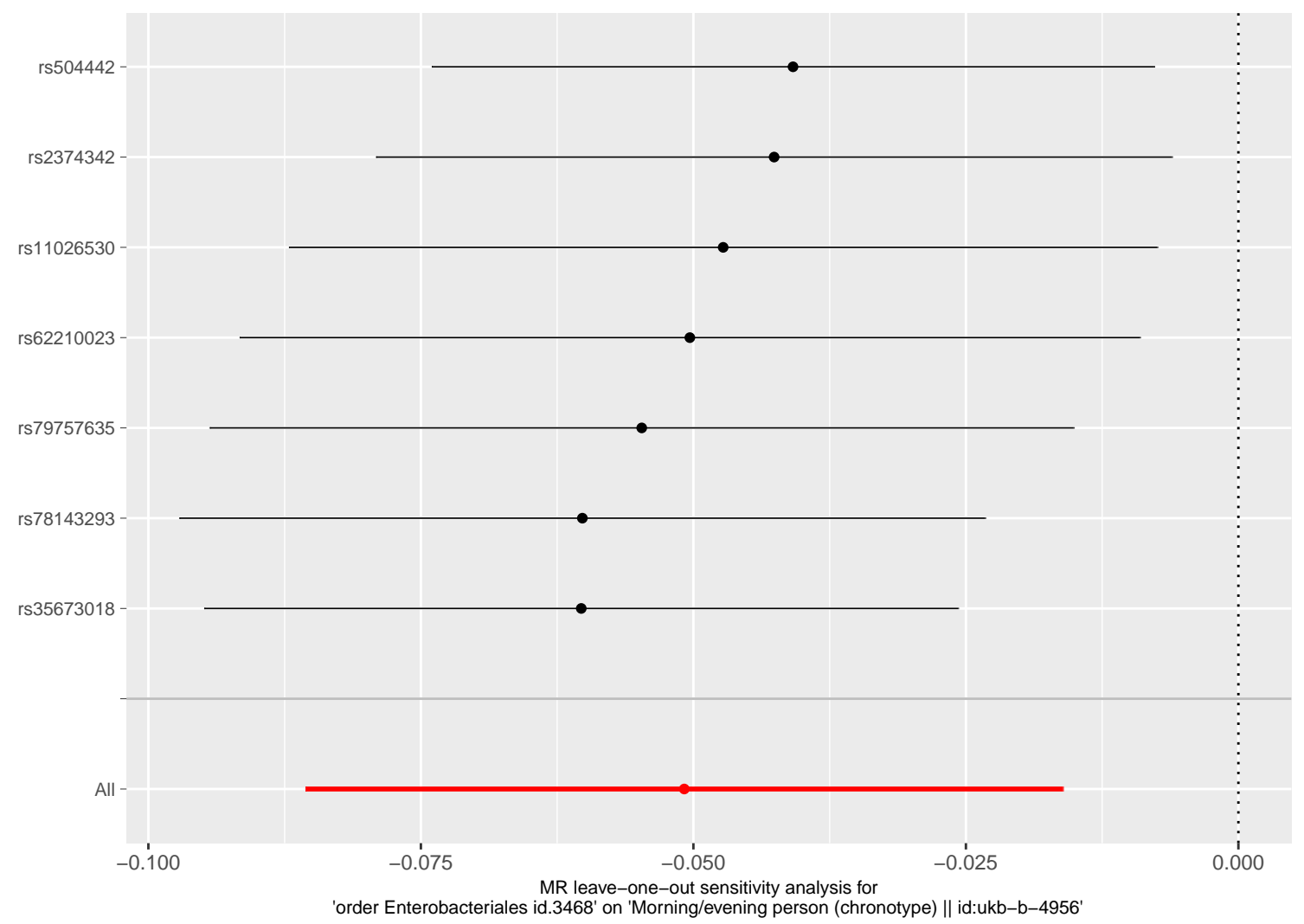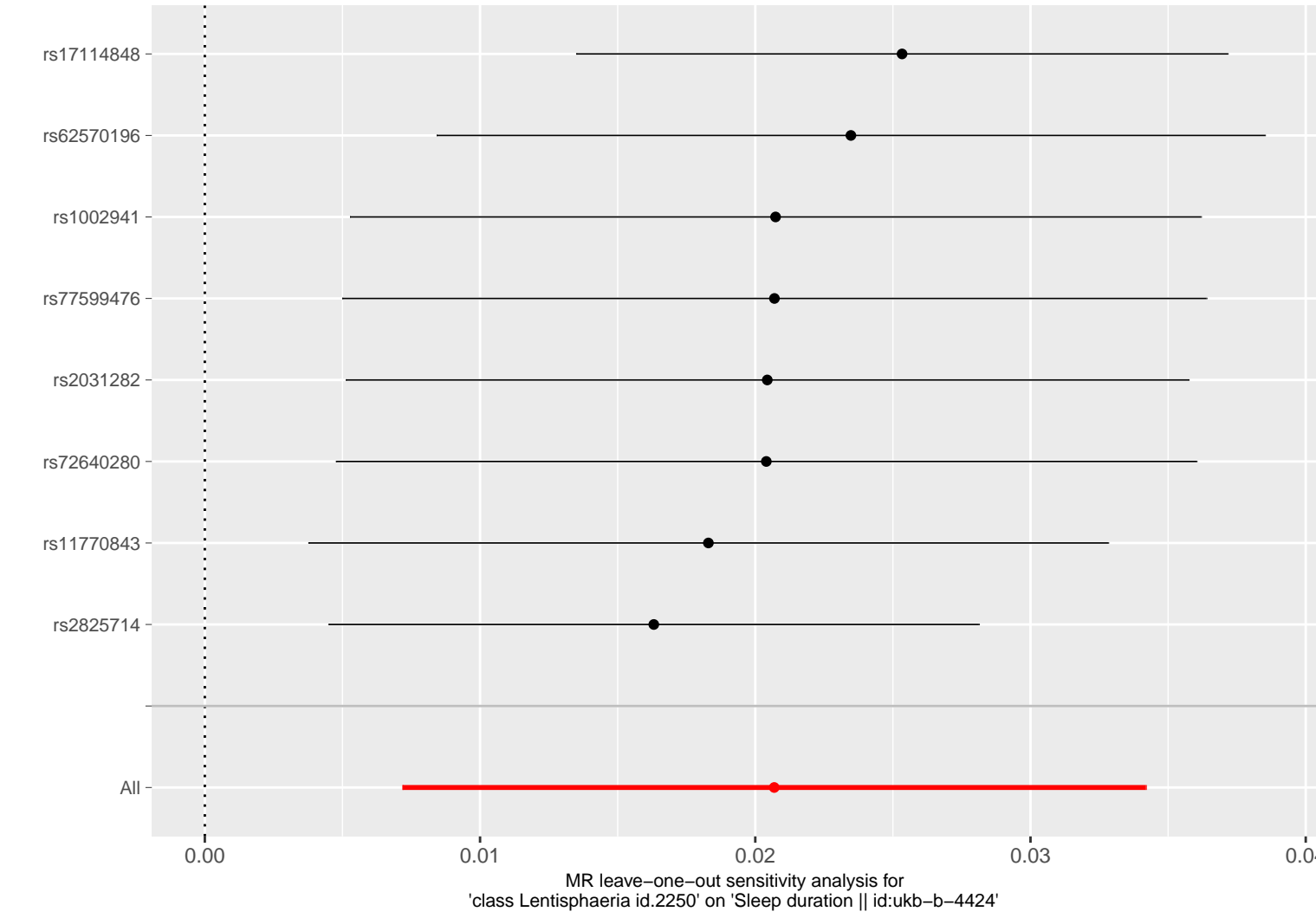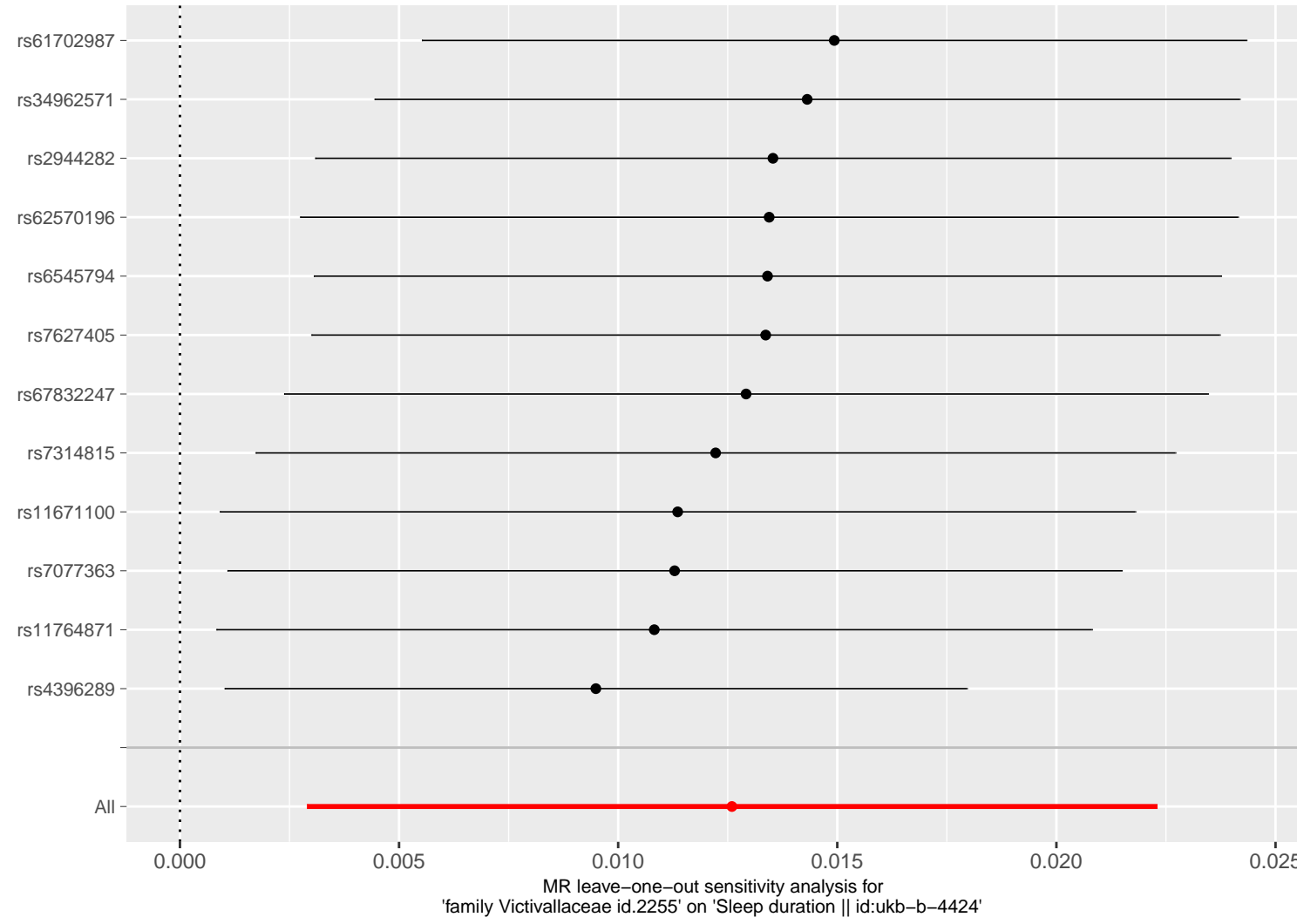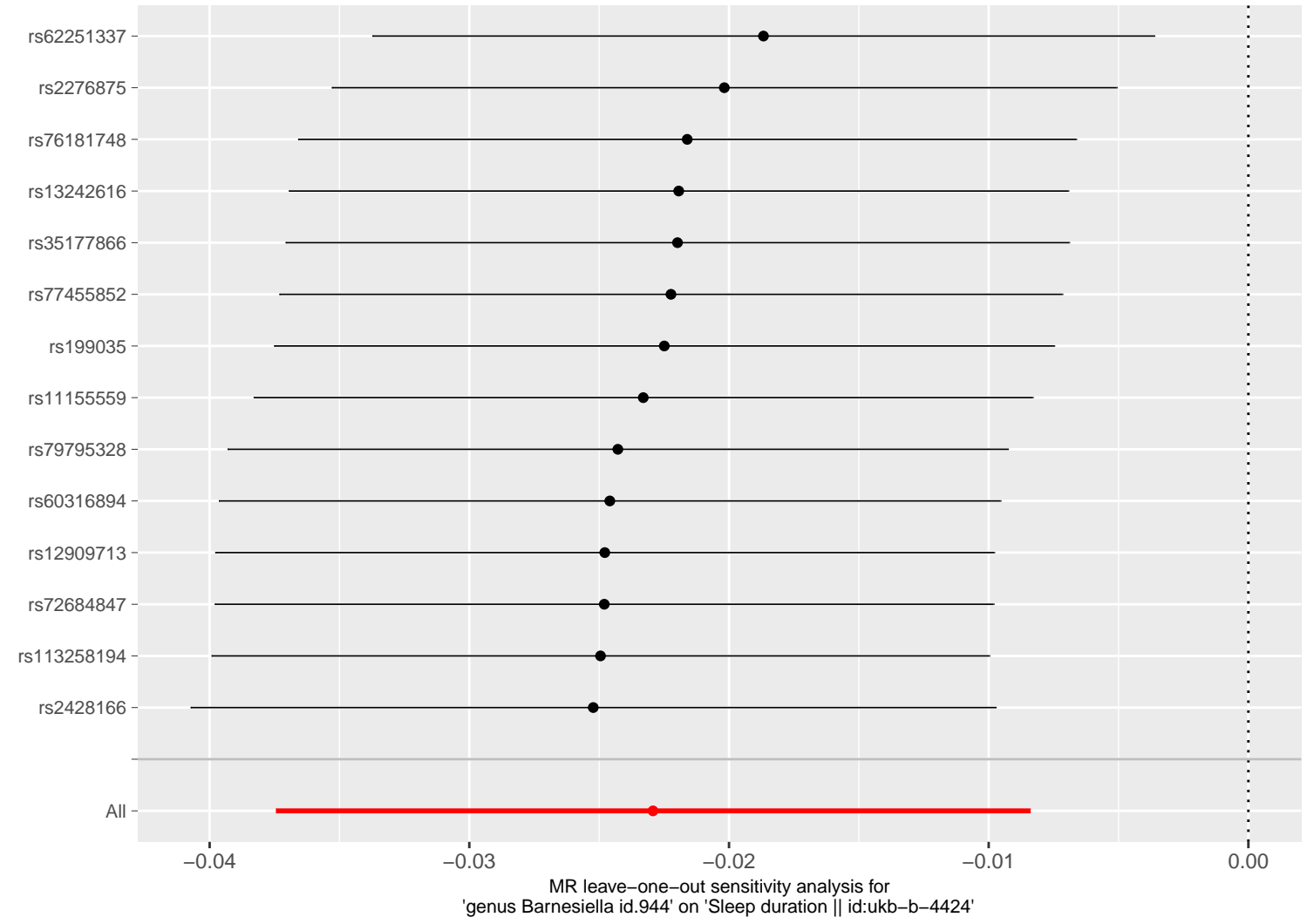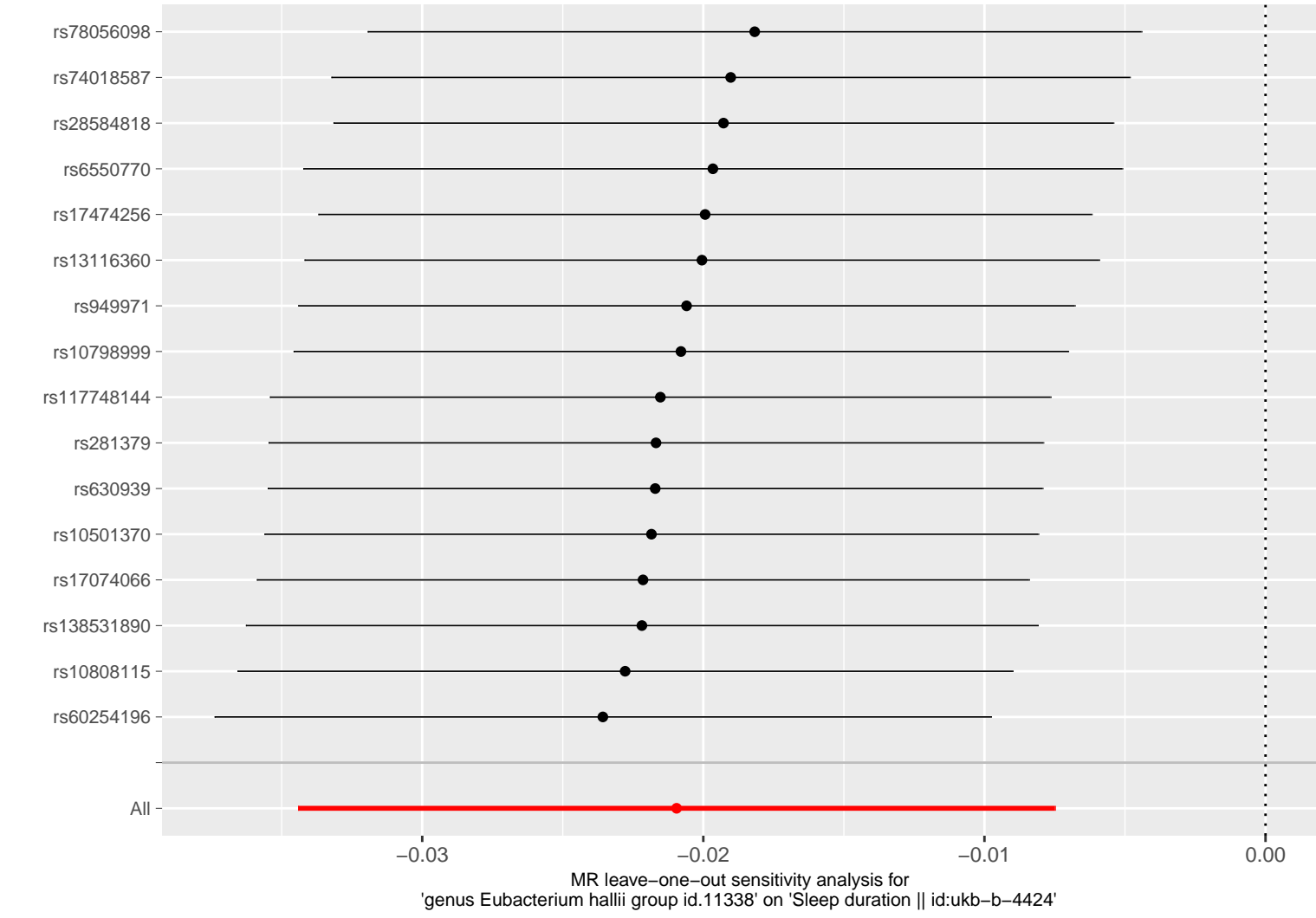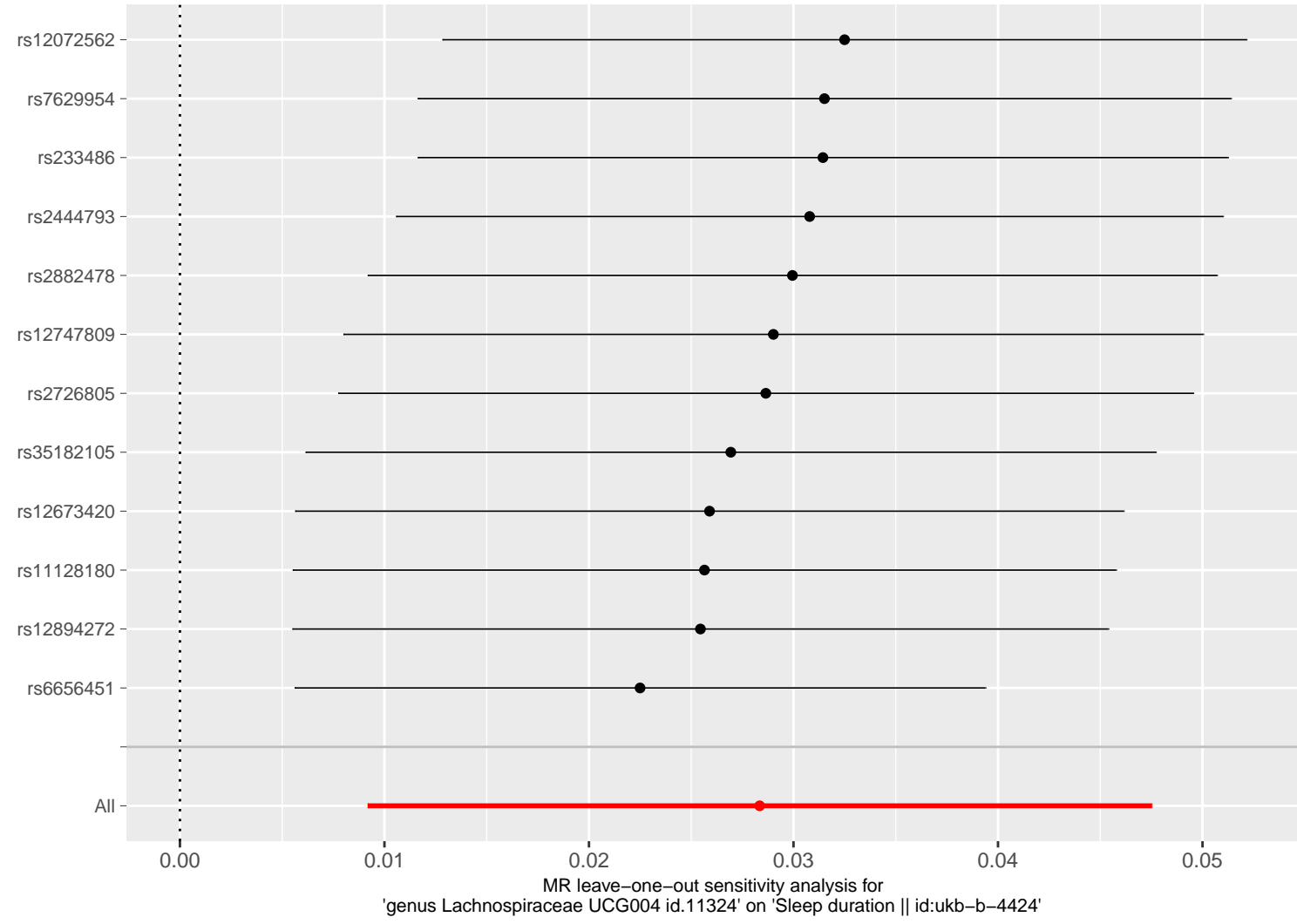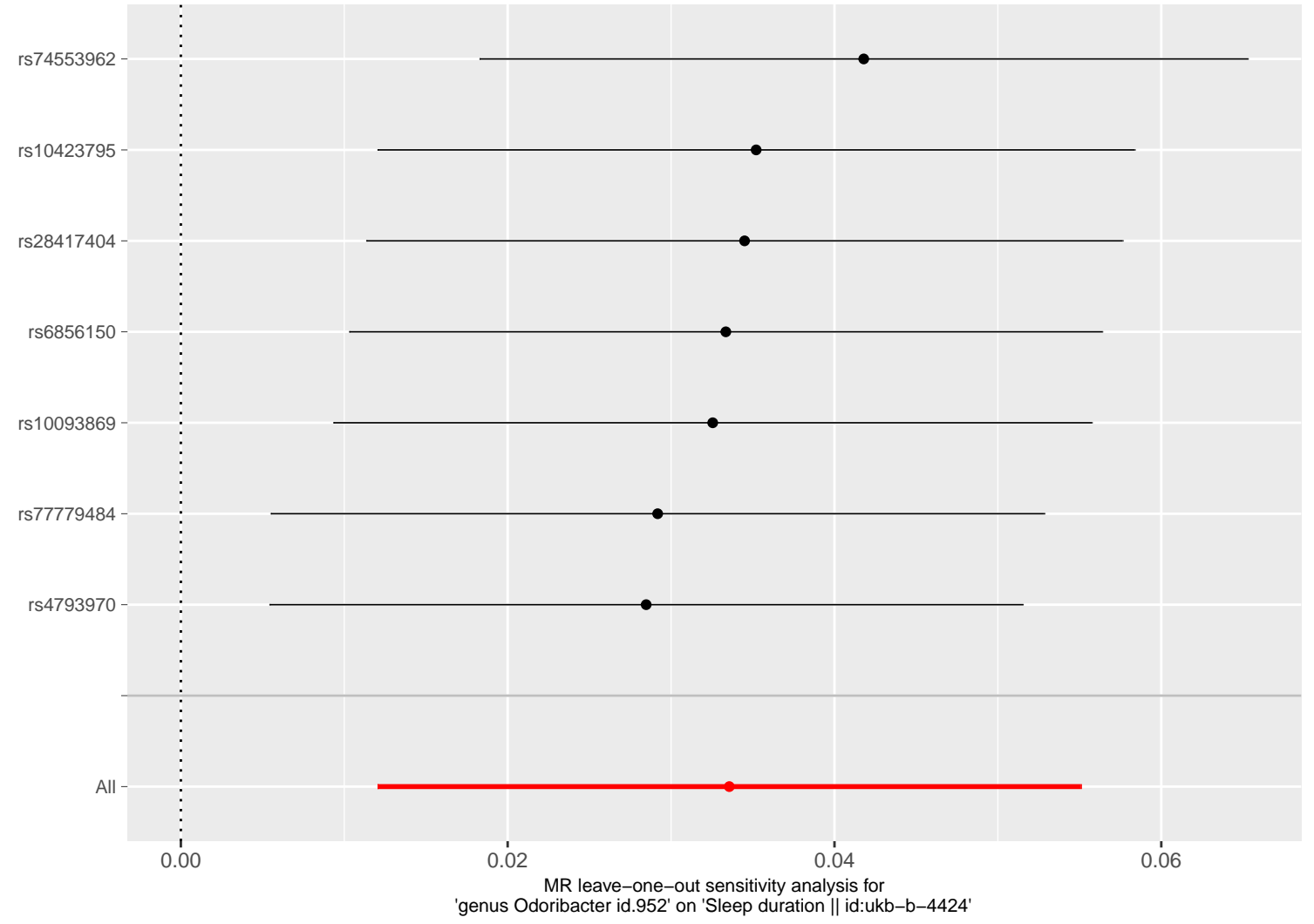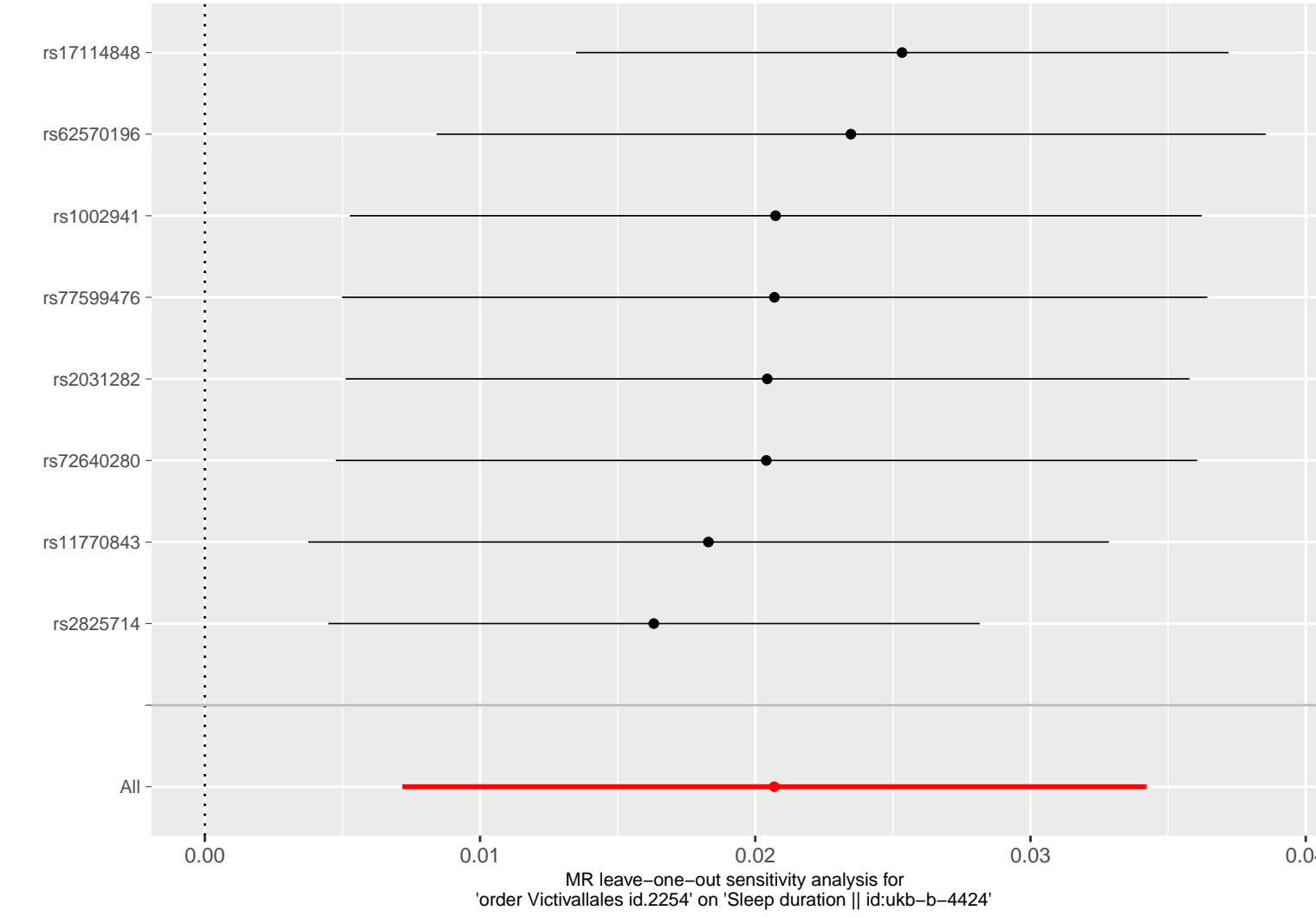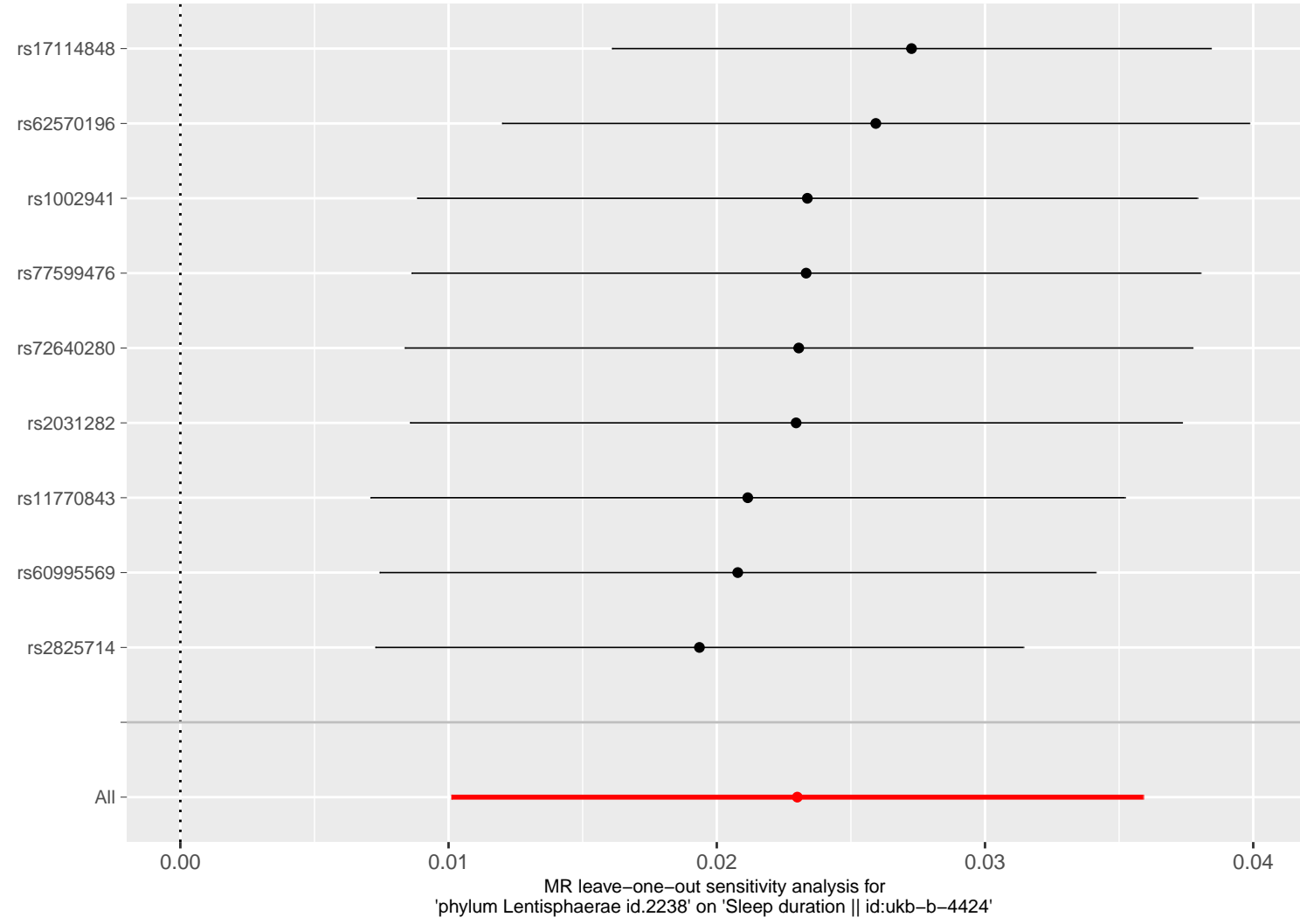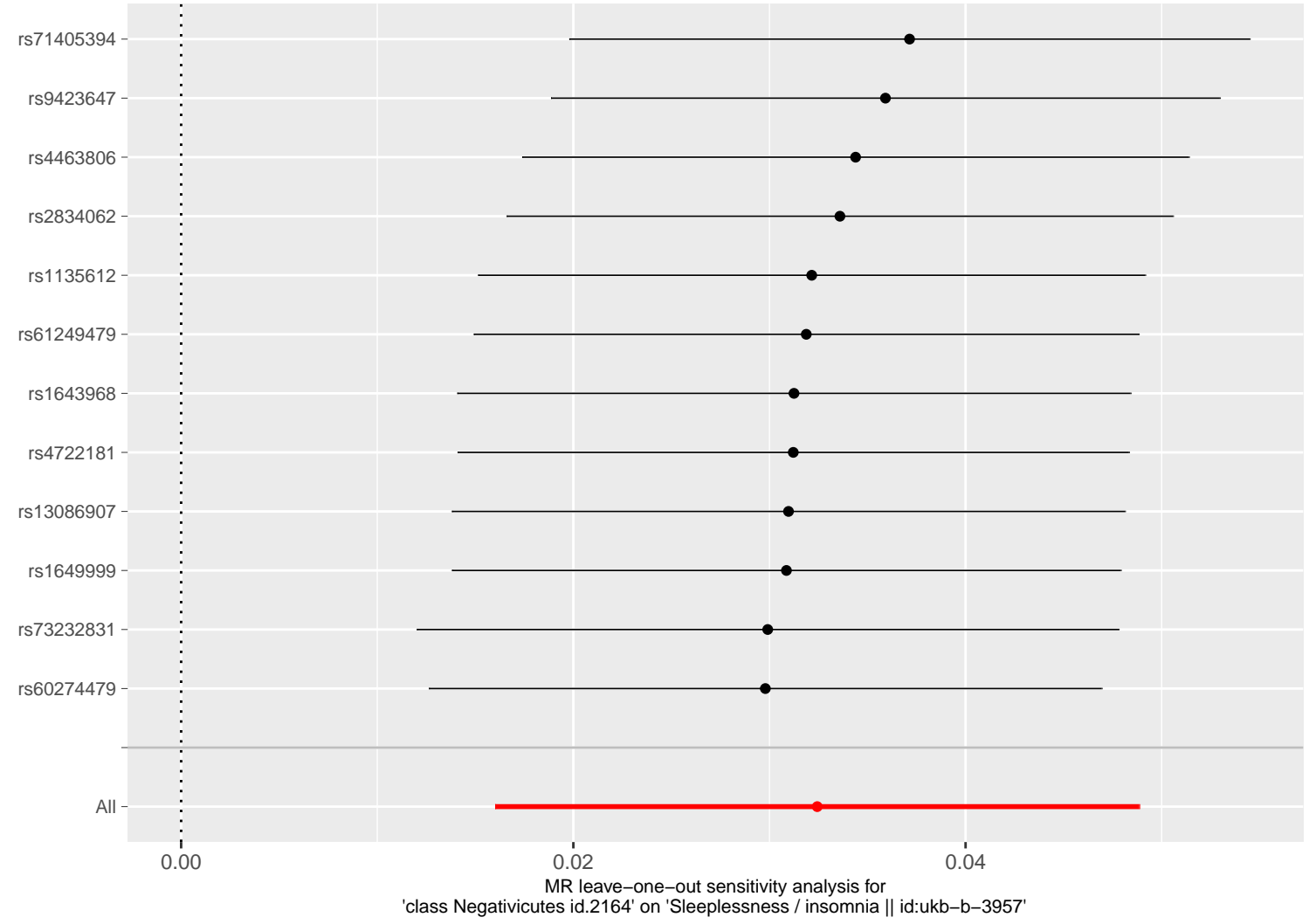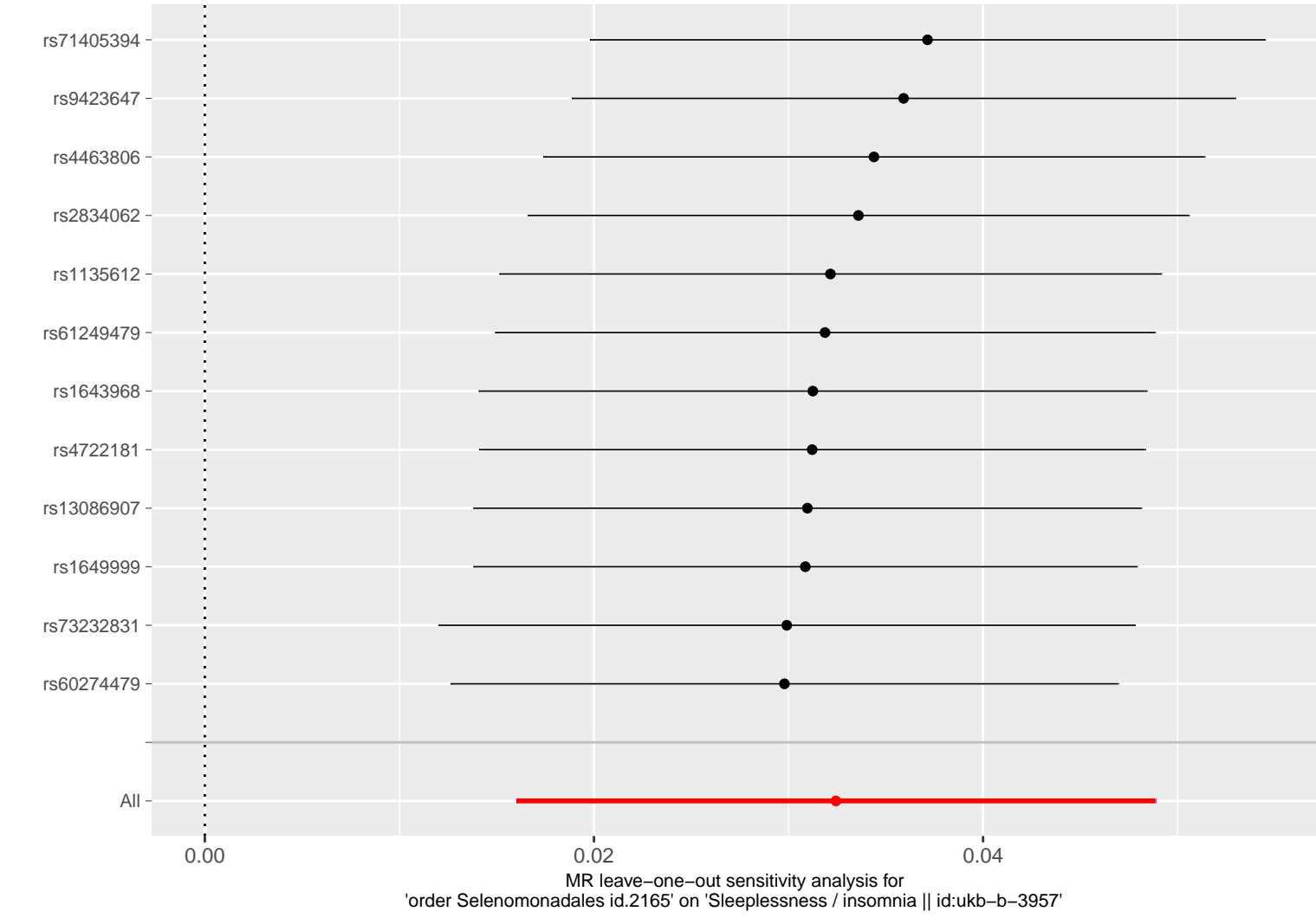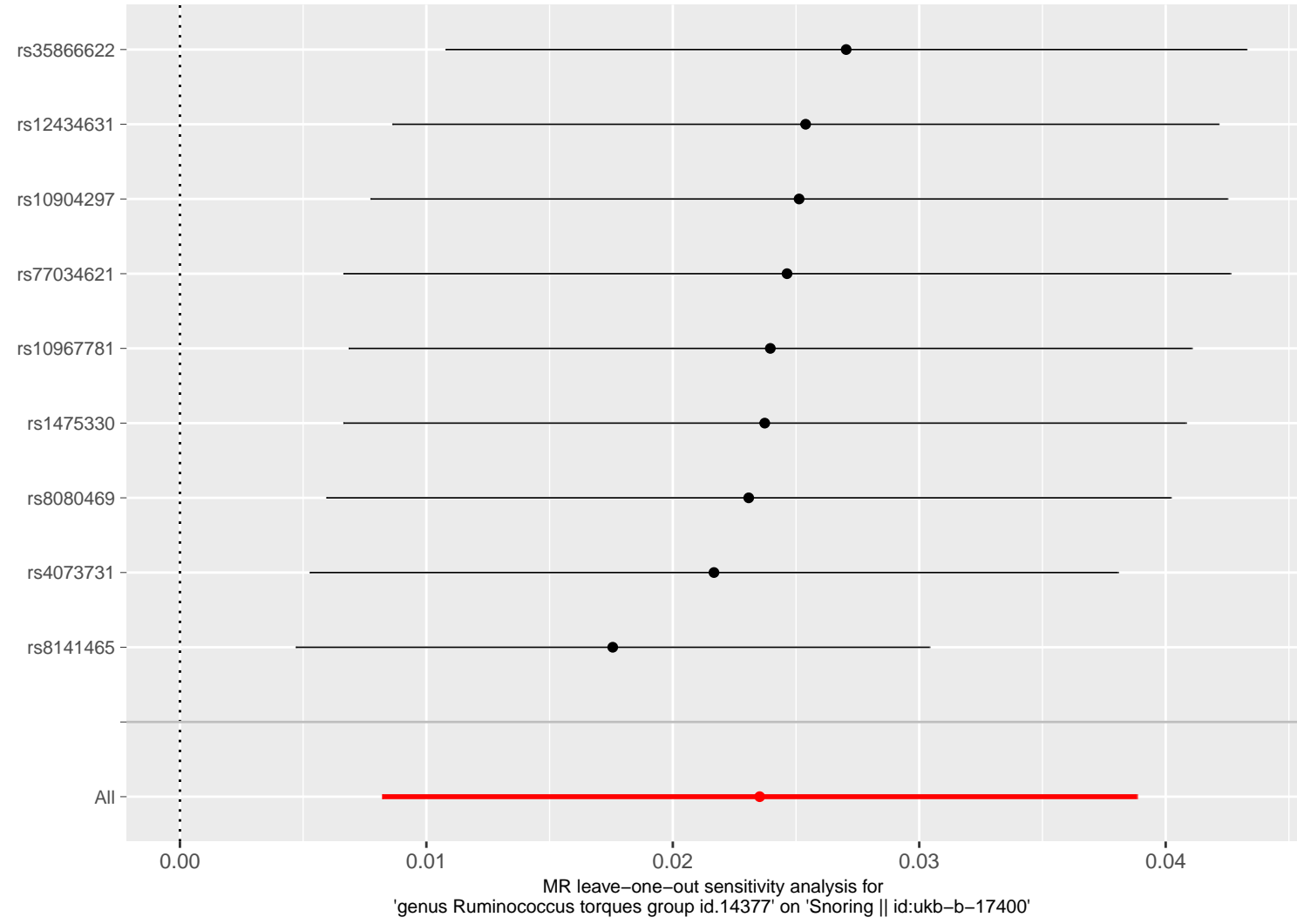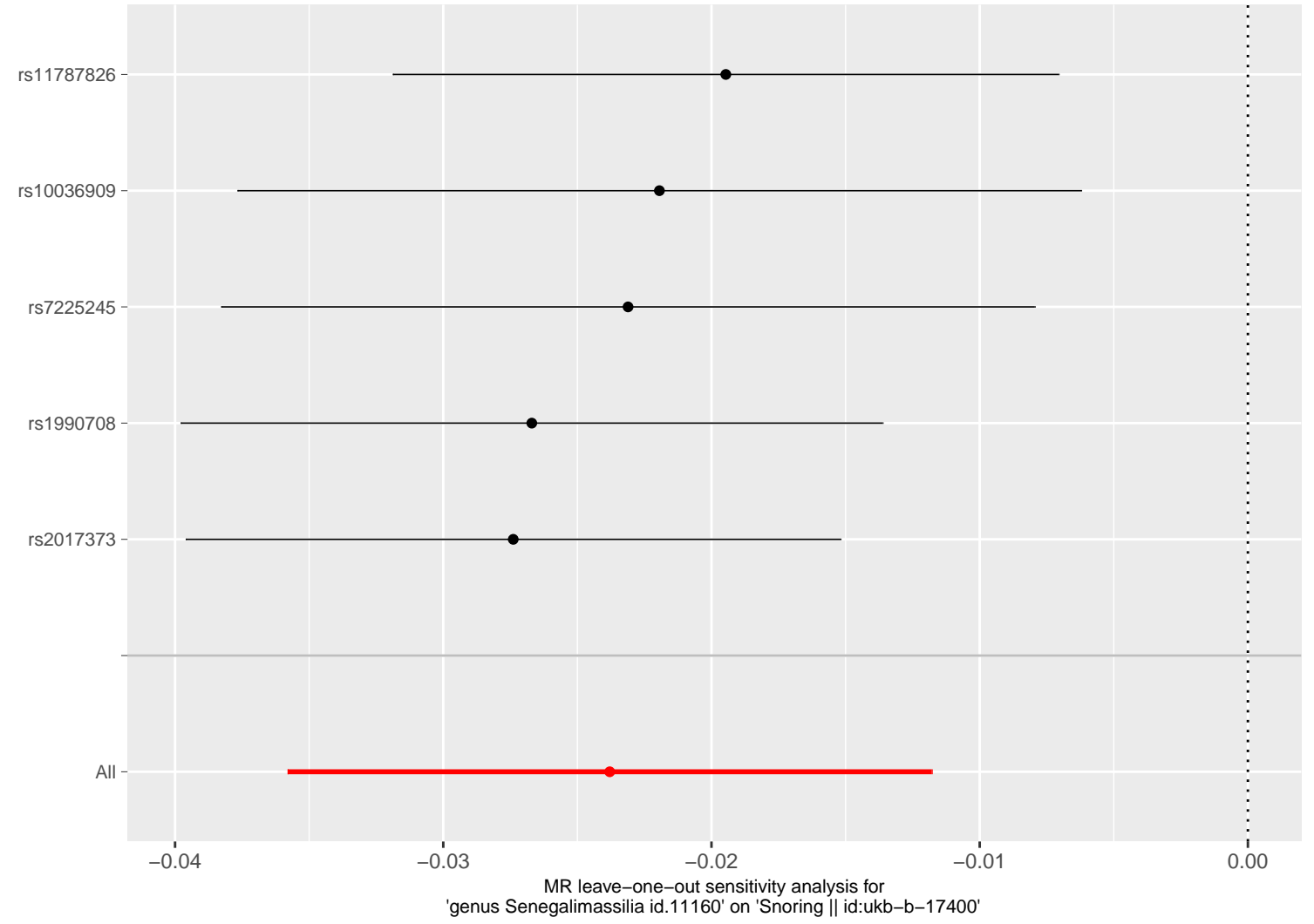

Supplement: Supplementary file 1 [file clockssleep-05-00037-s001.zip › Figure S1.pdf]

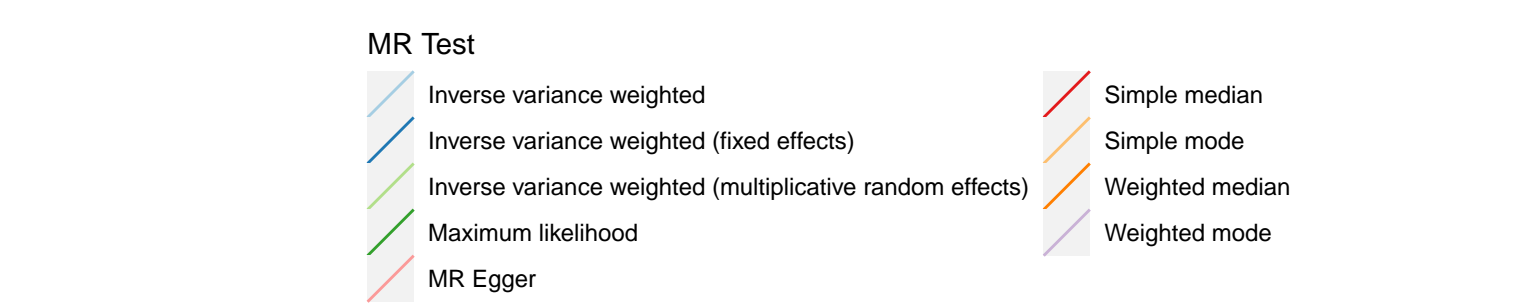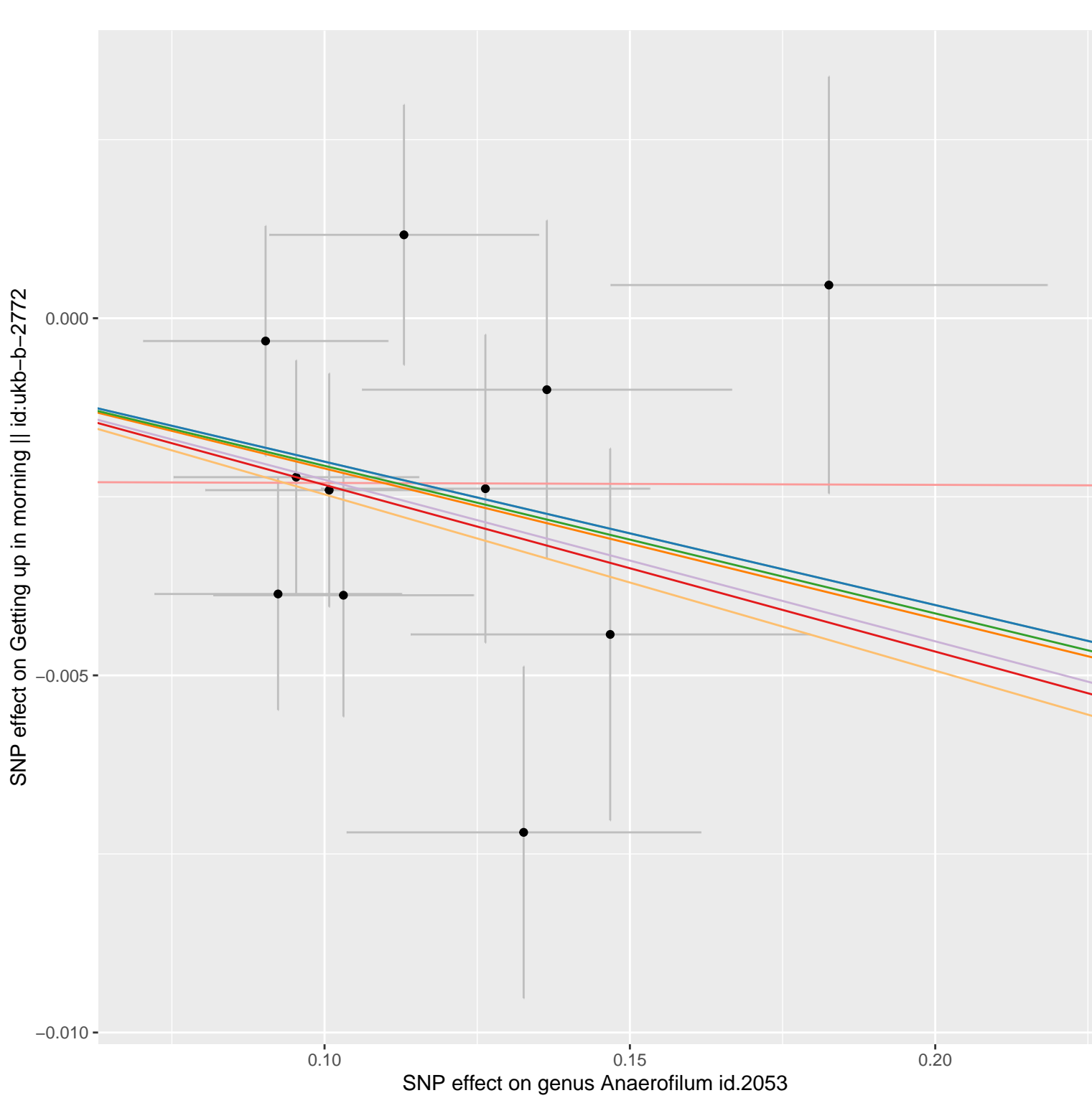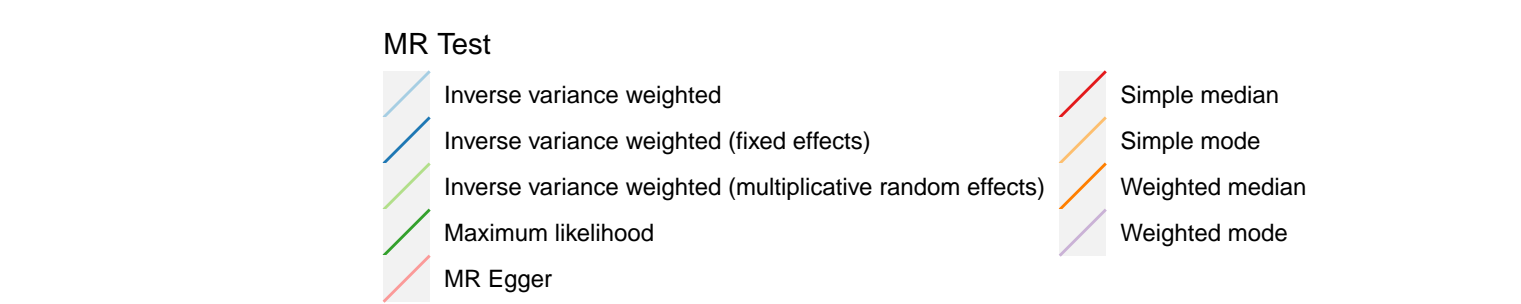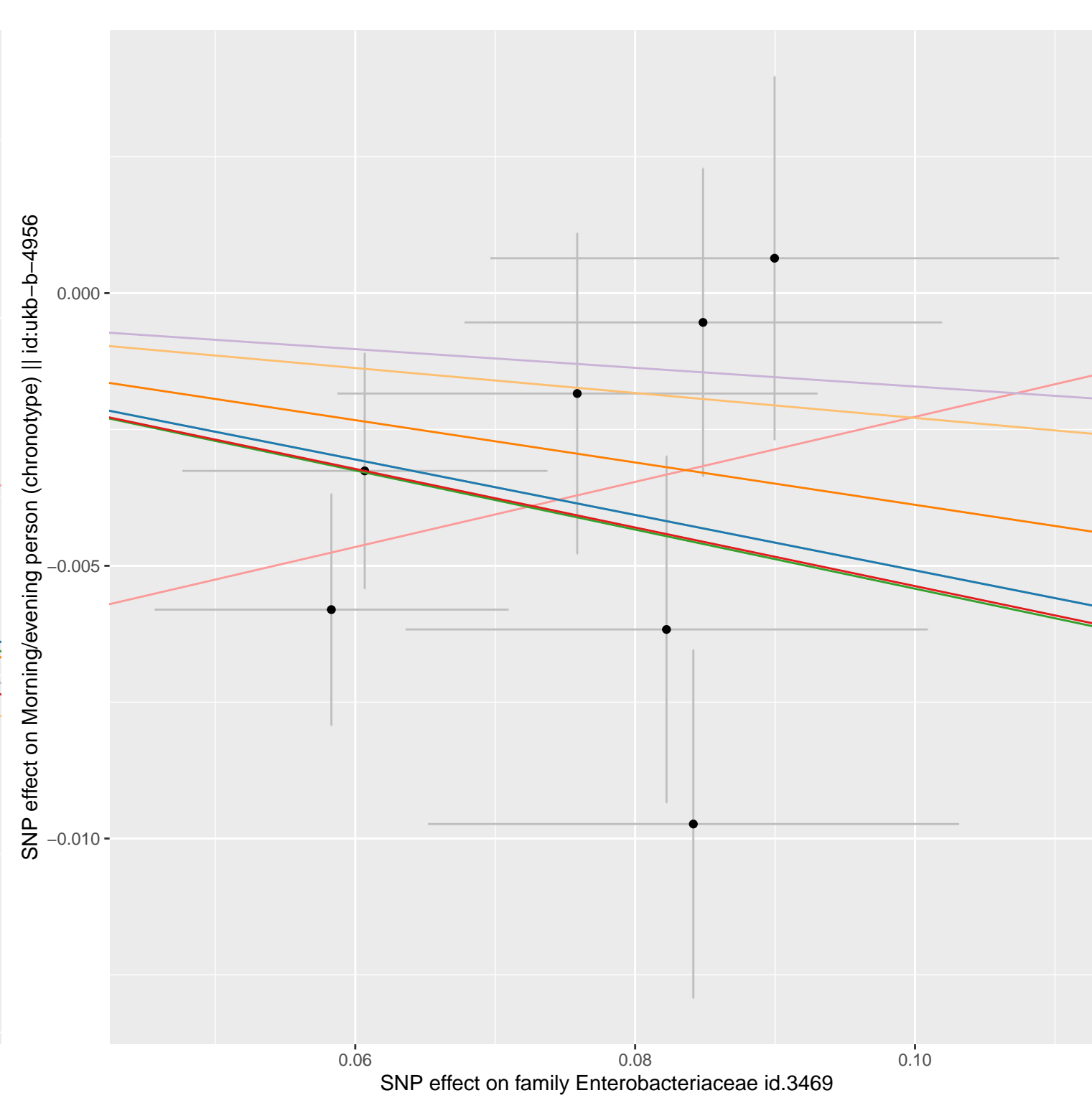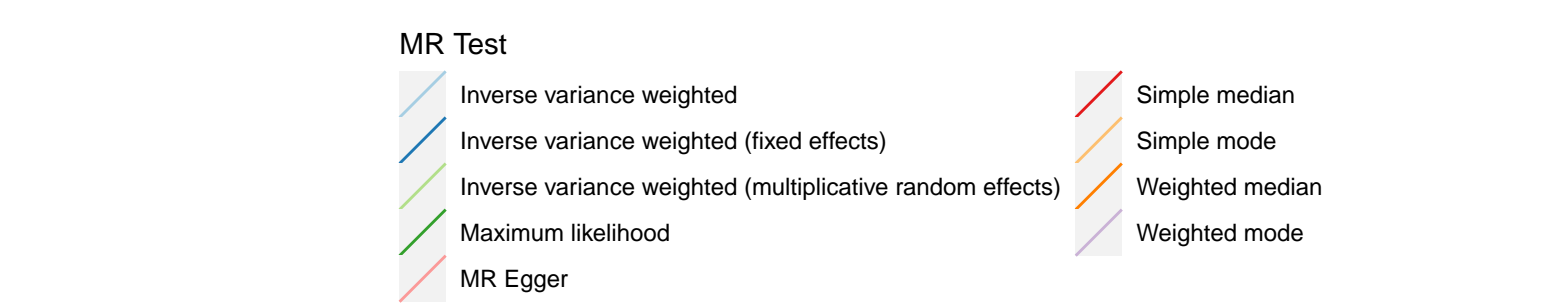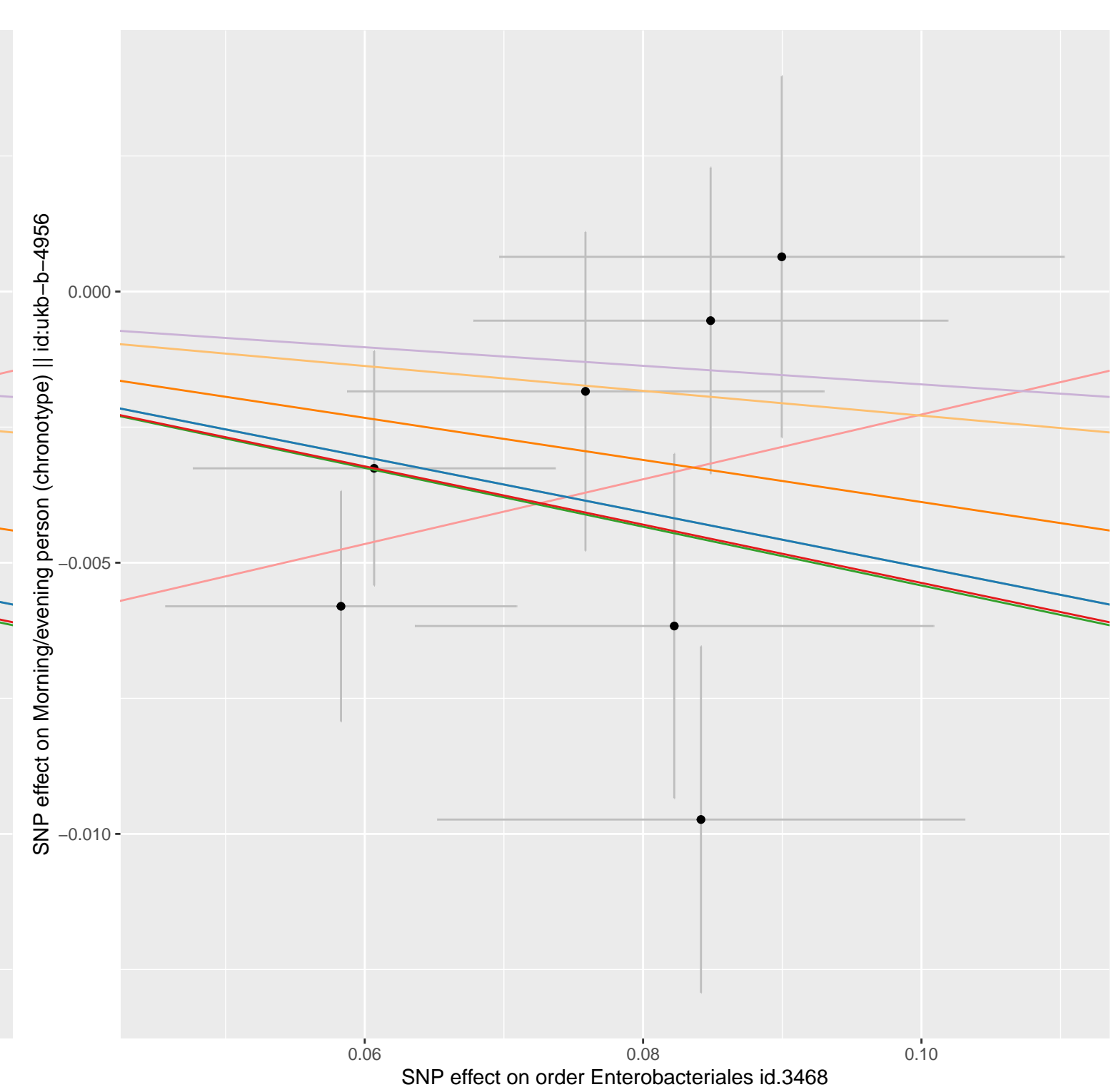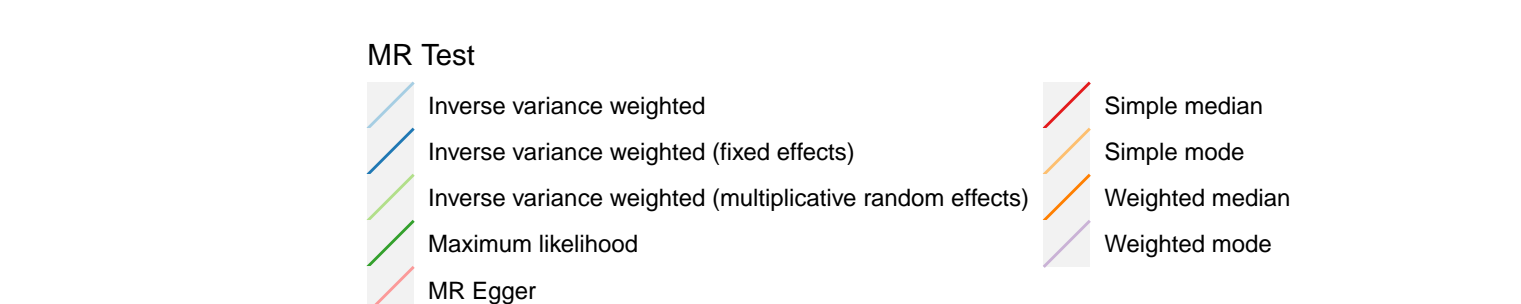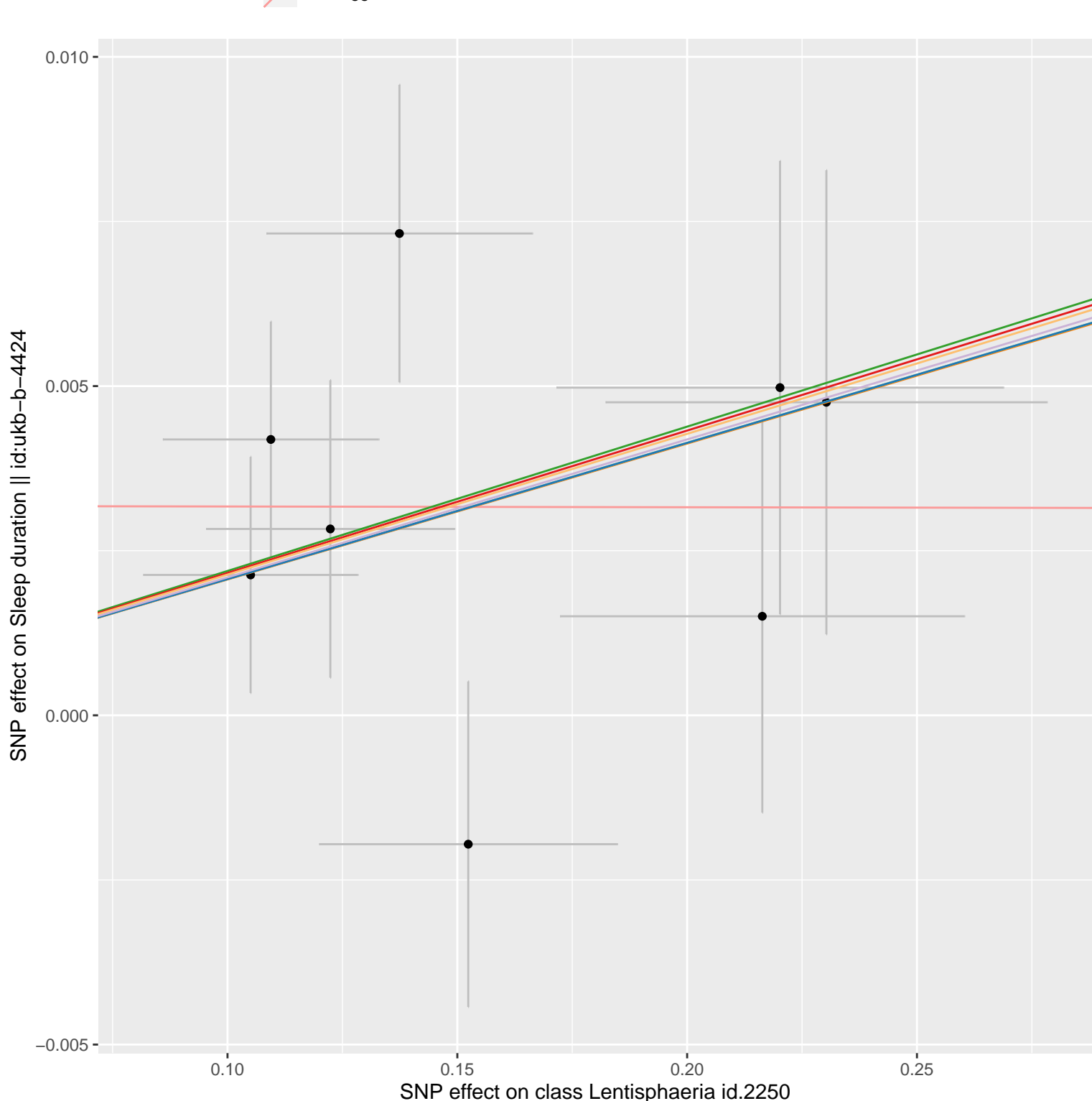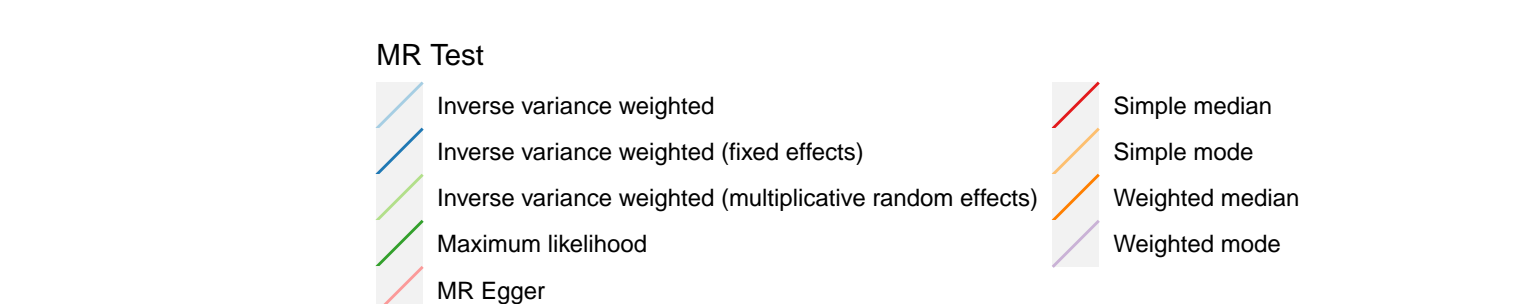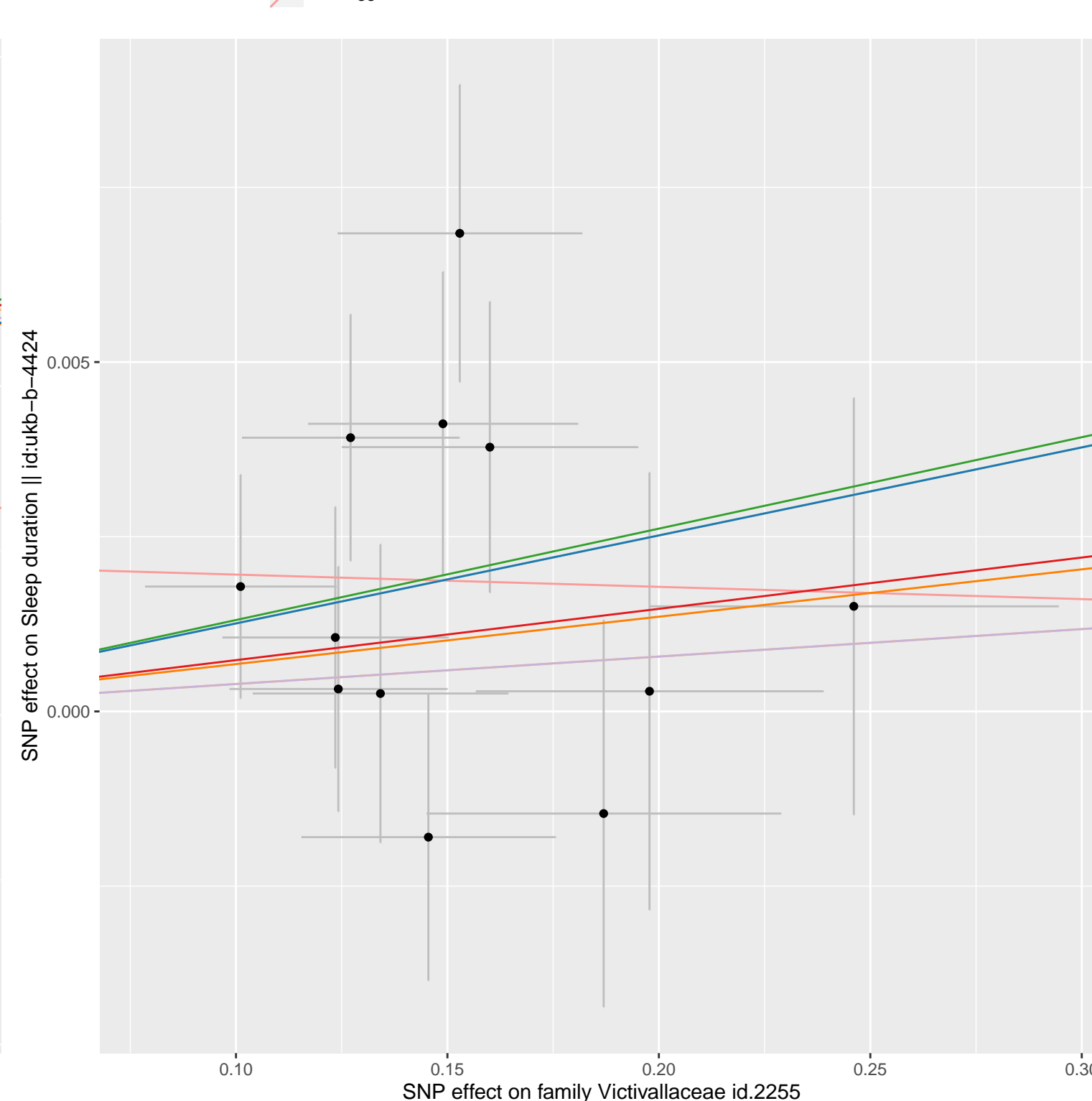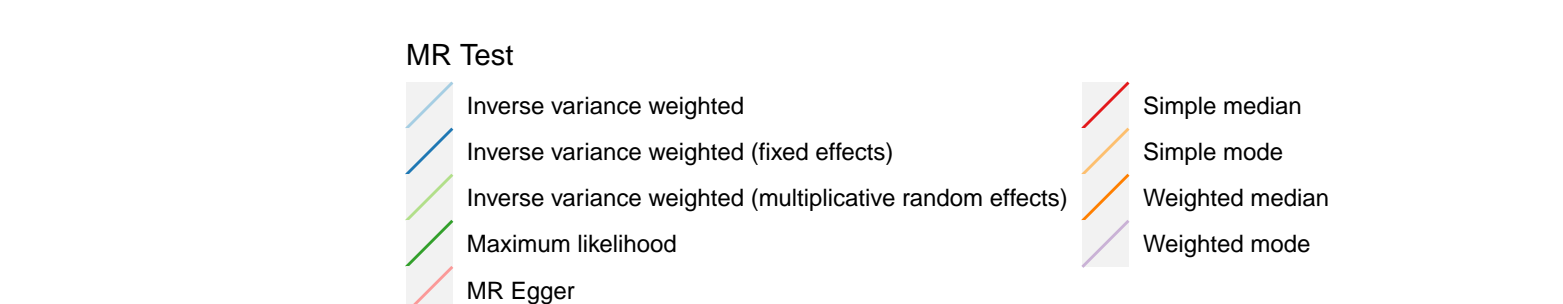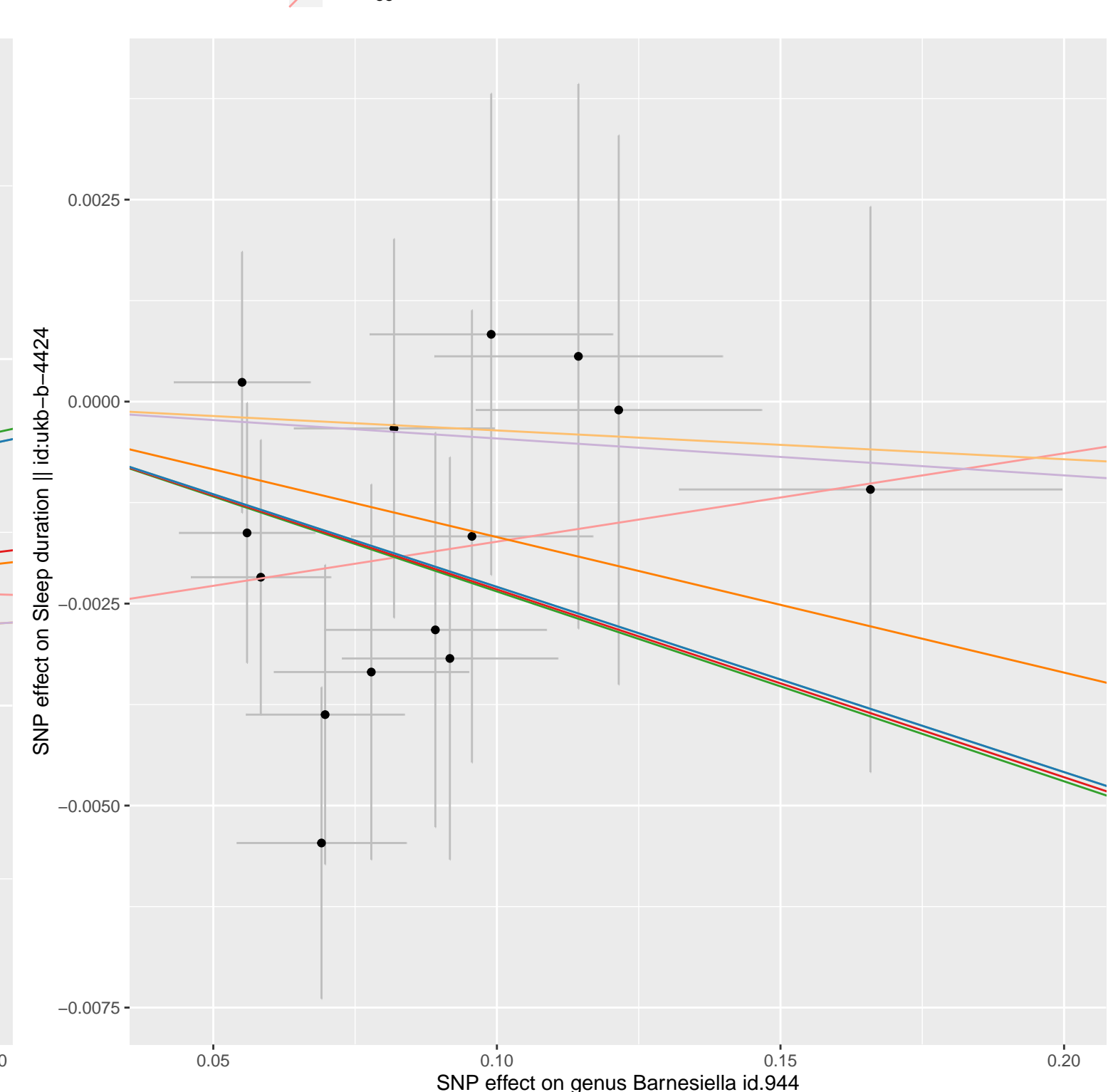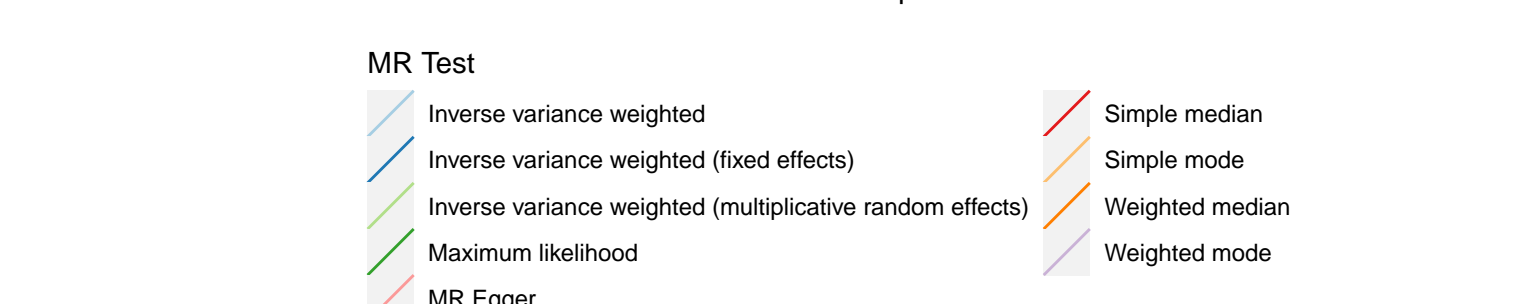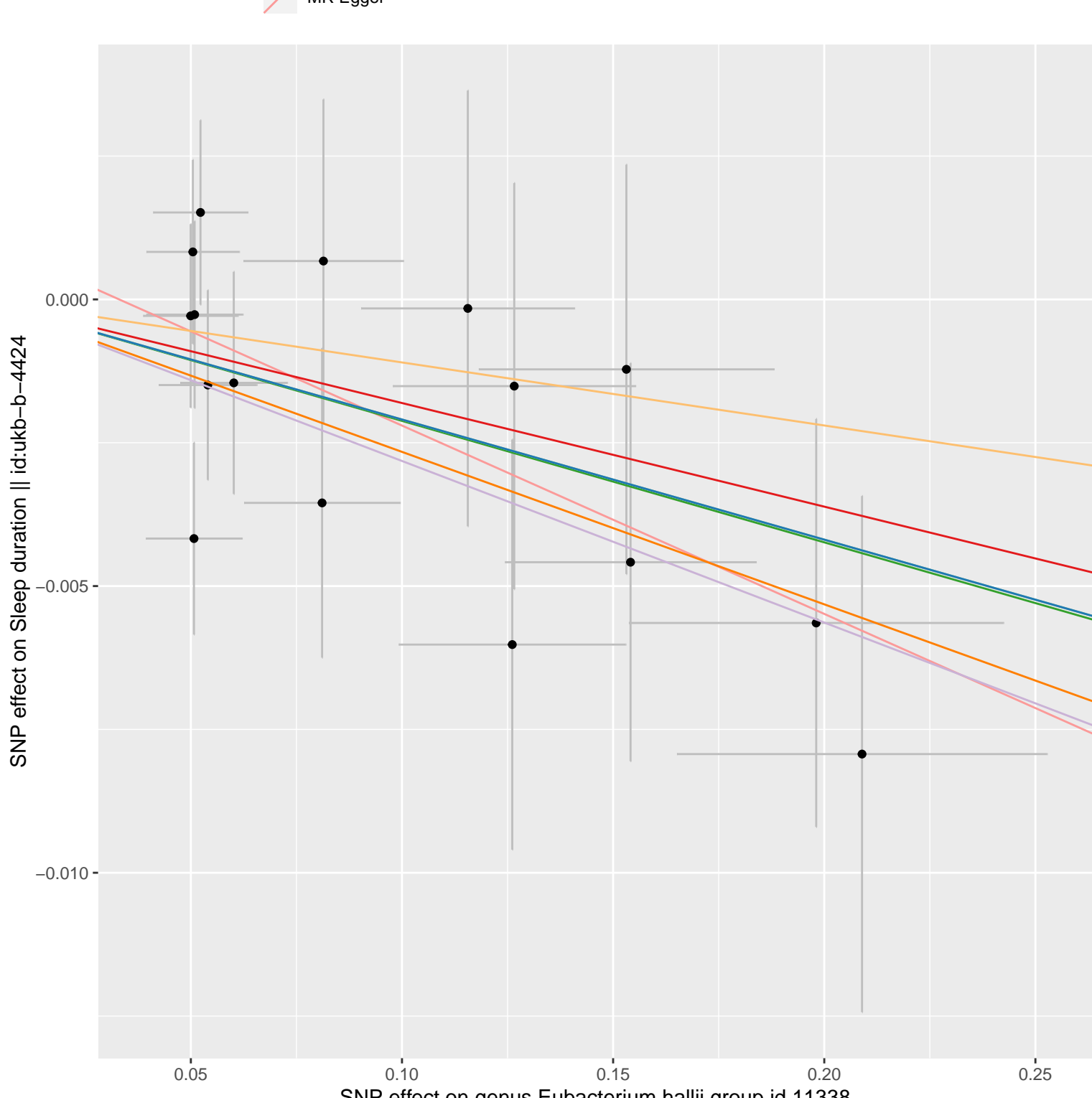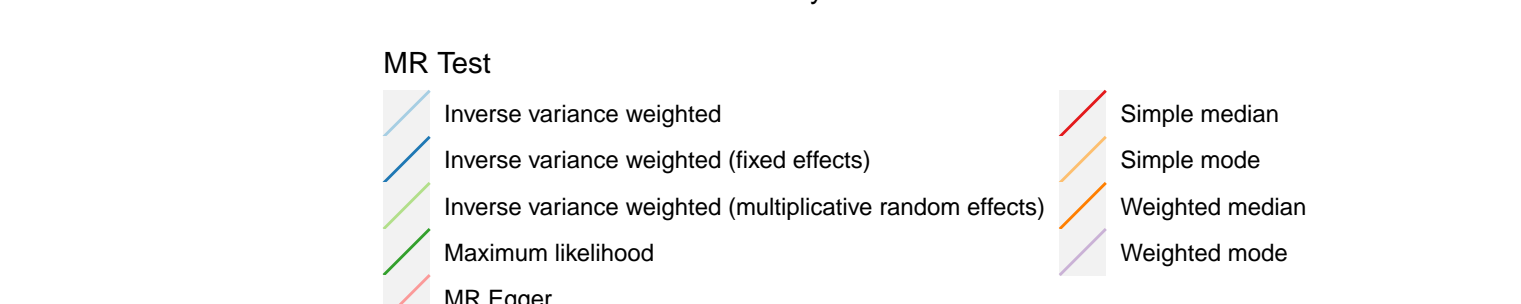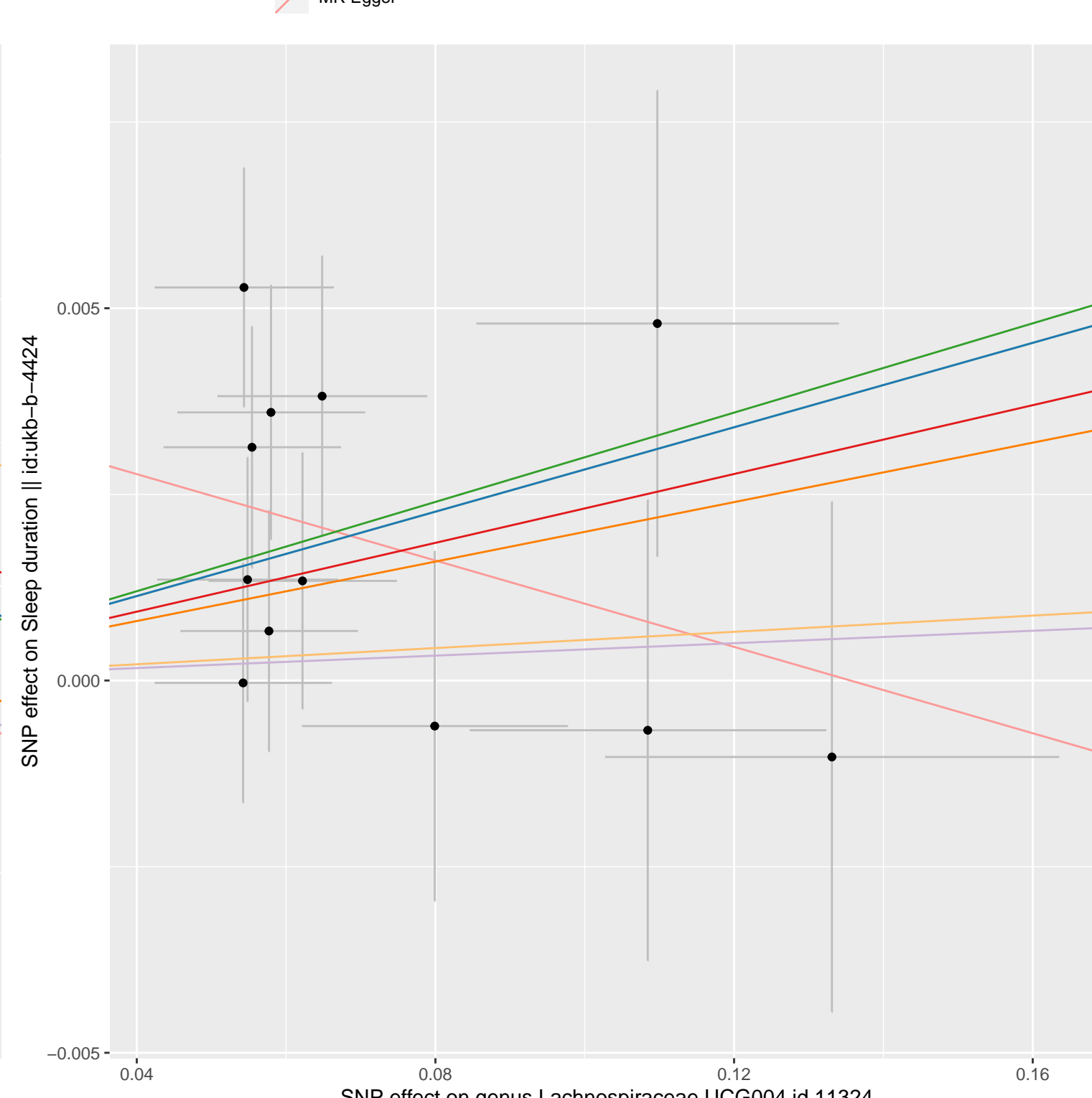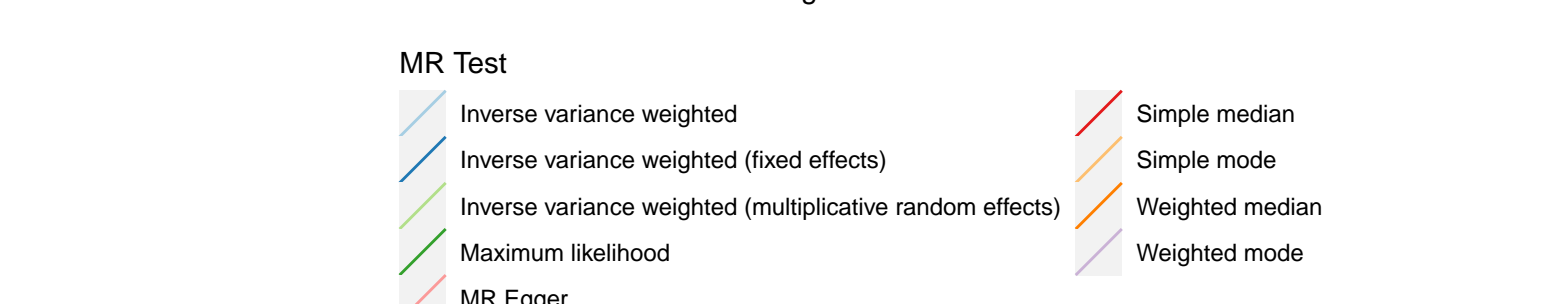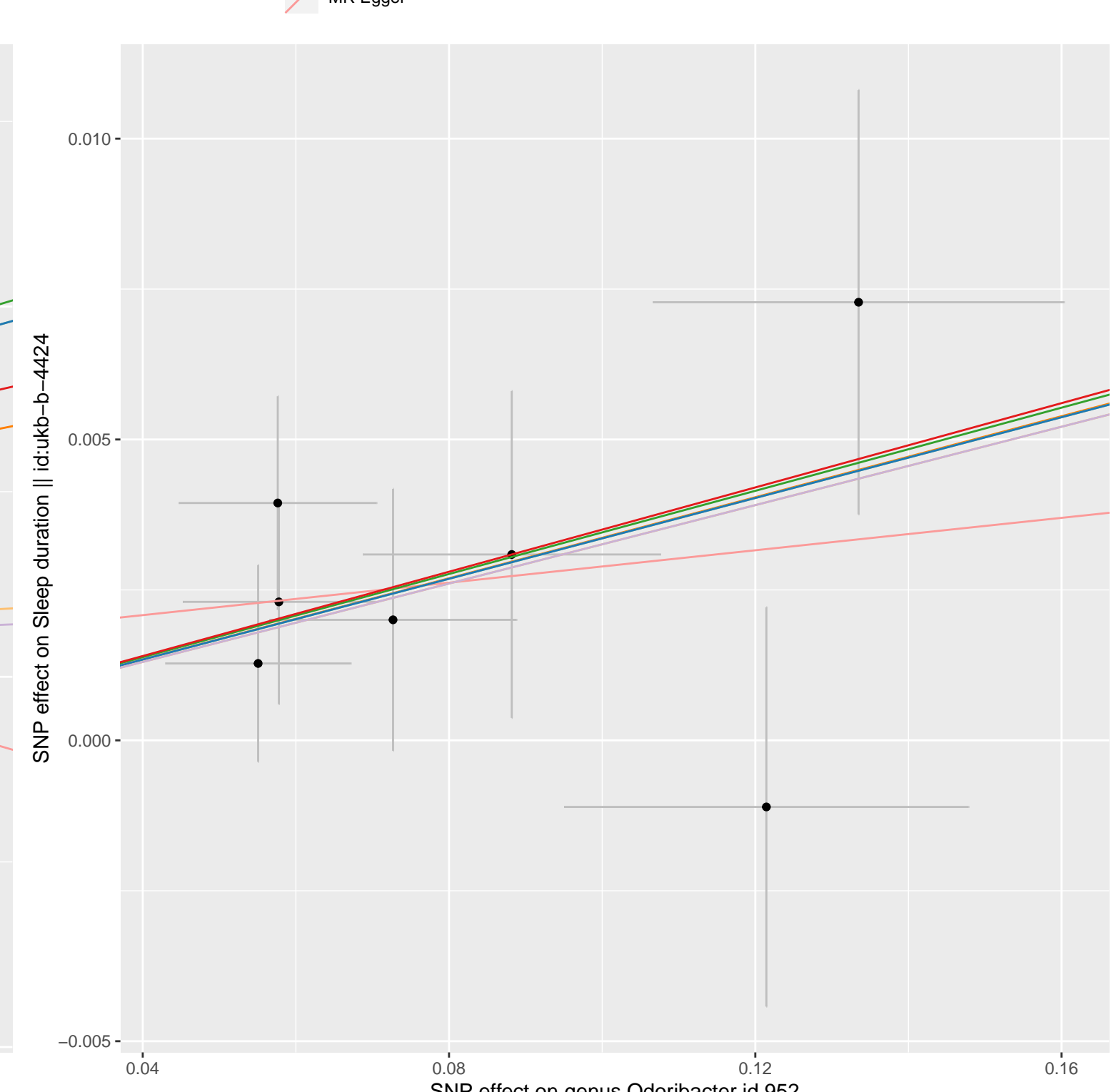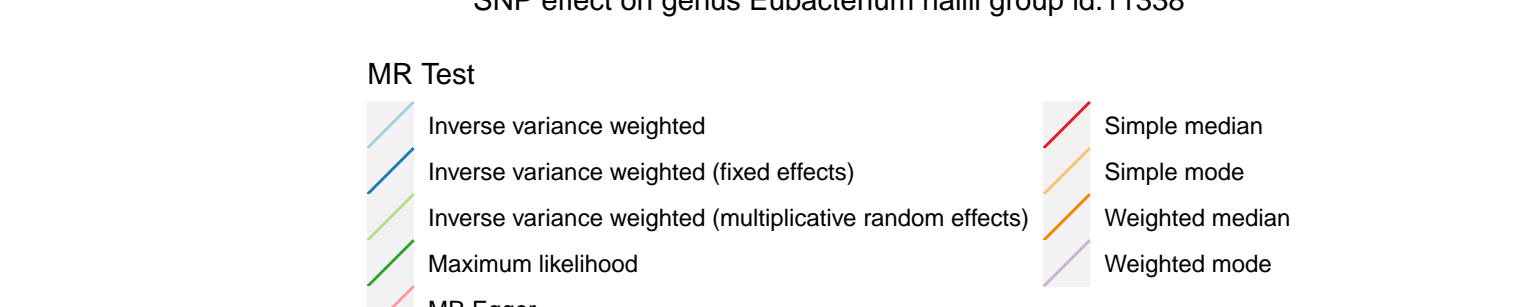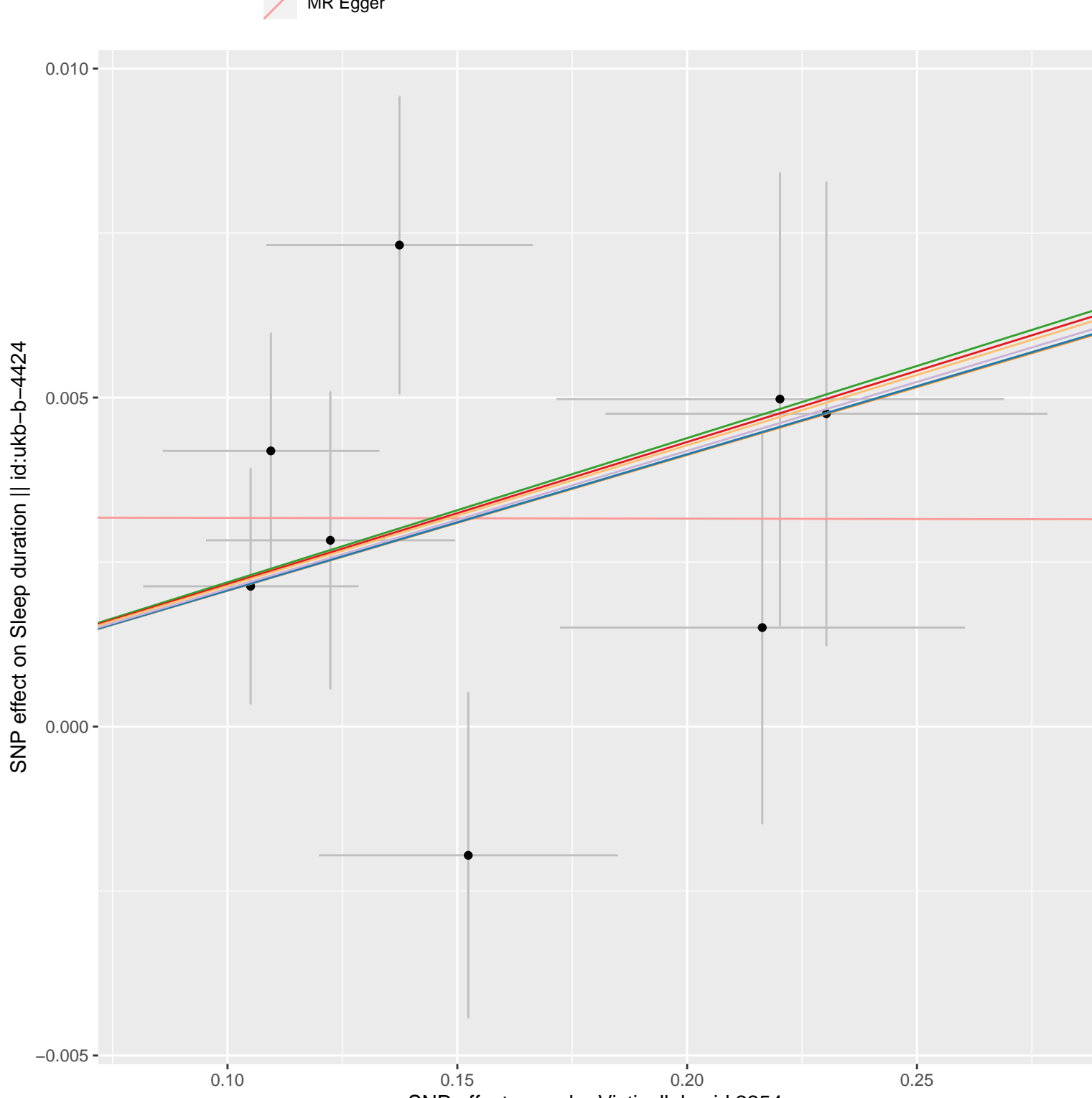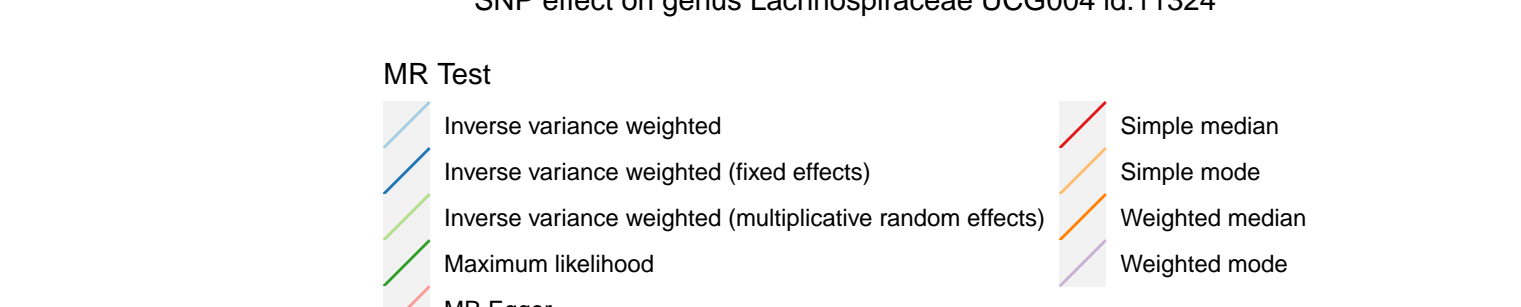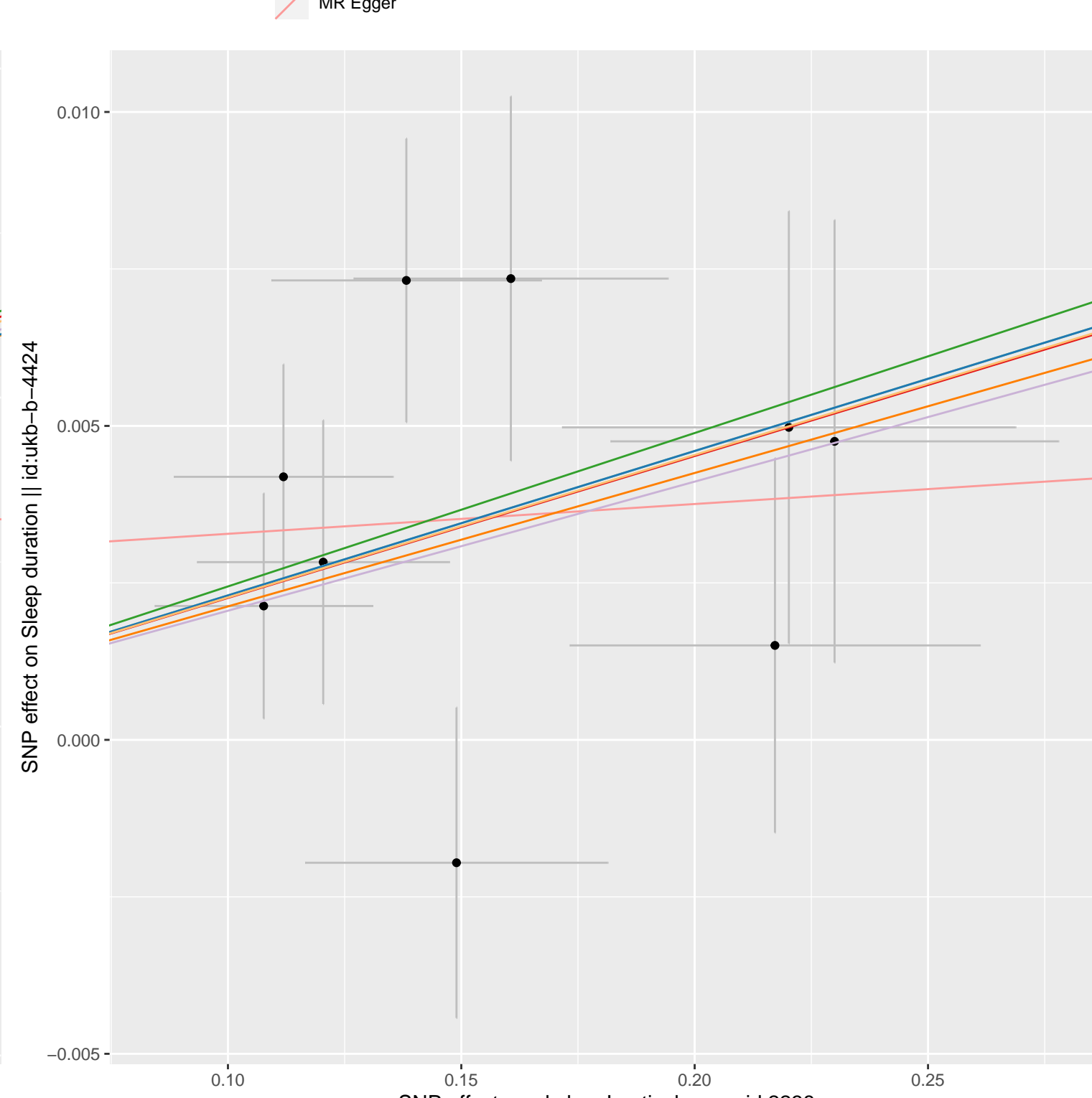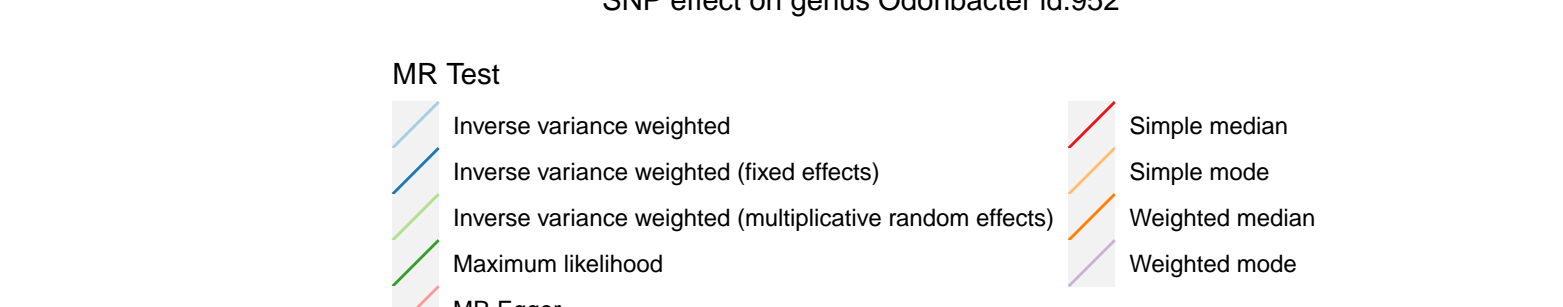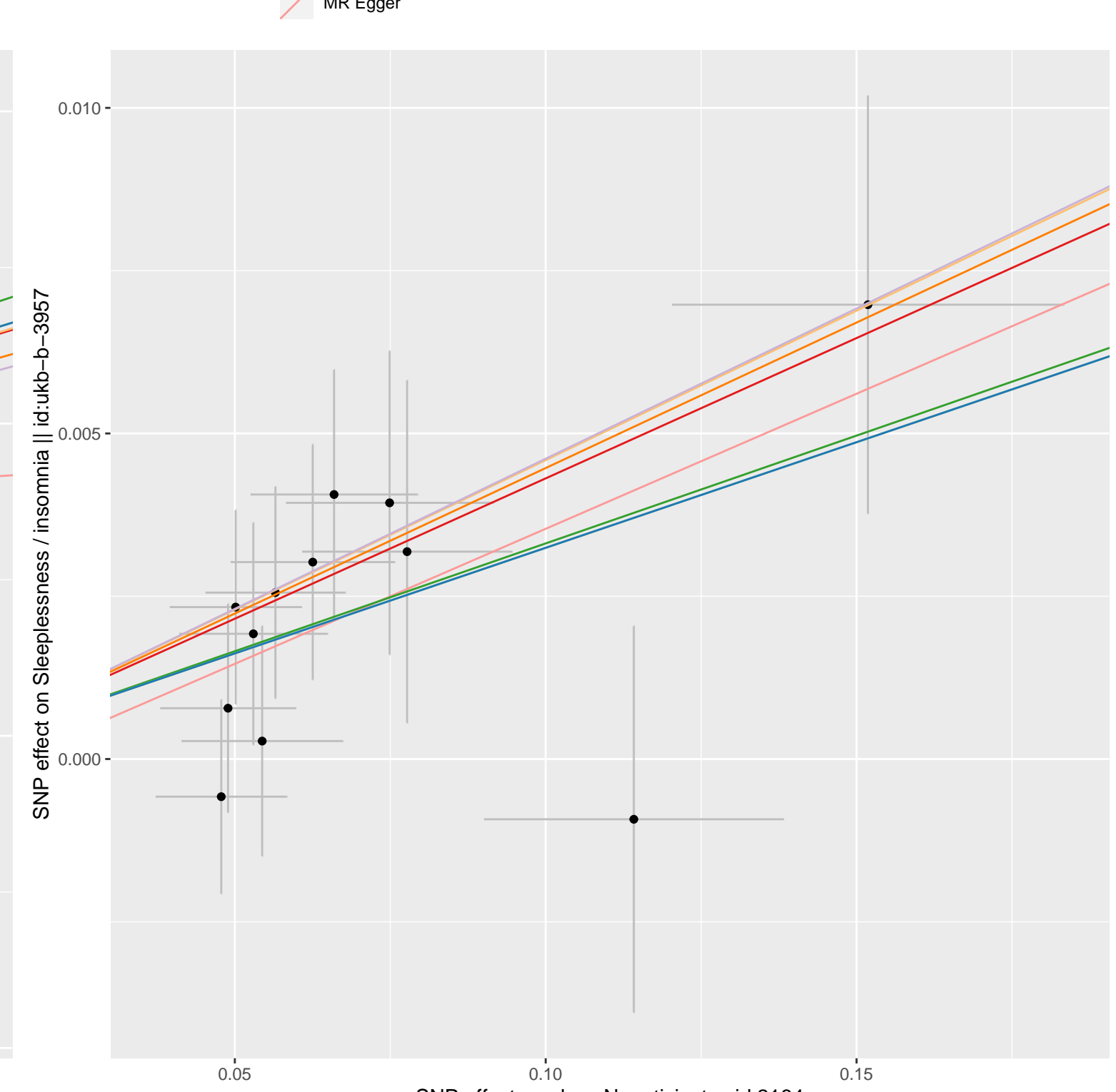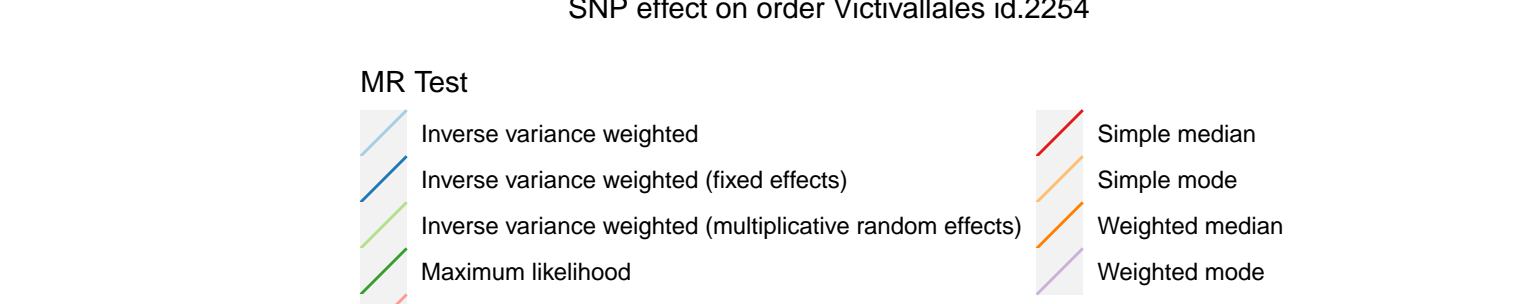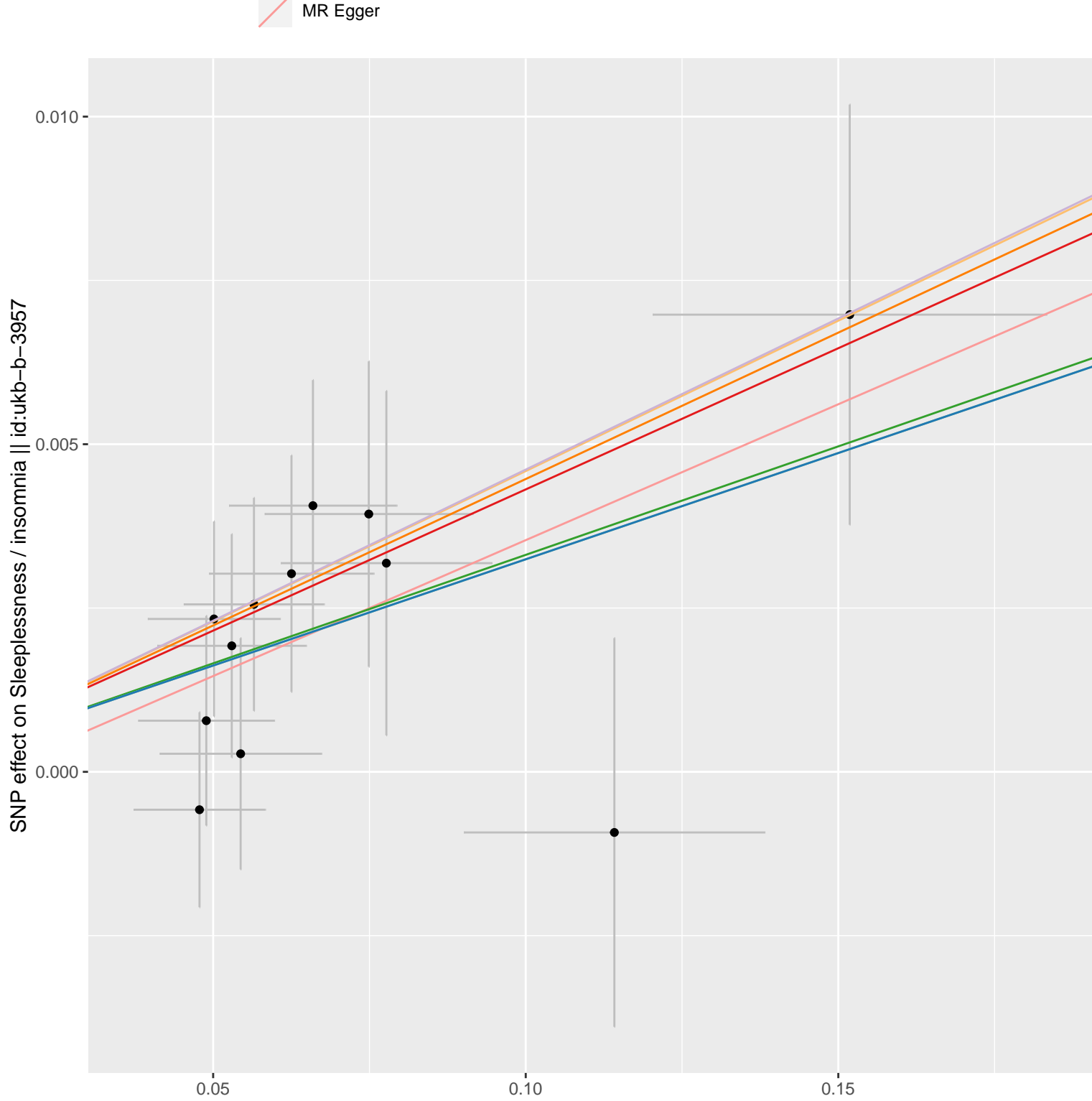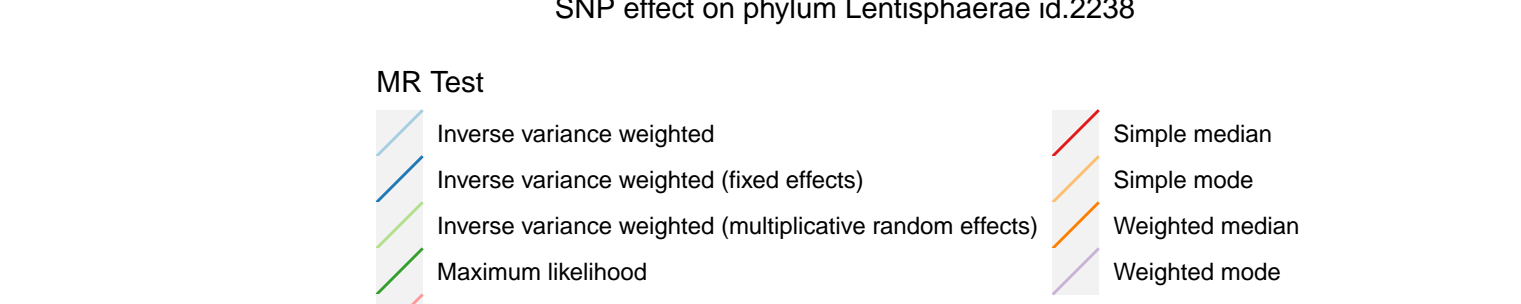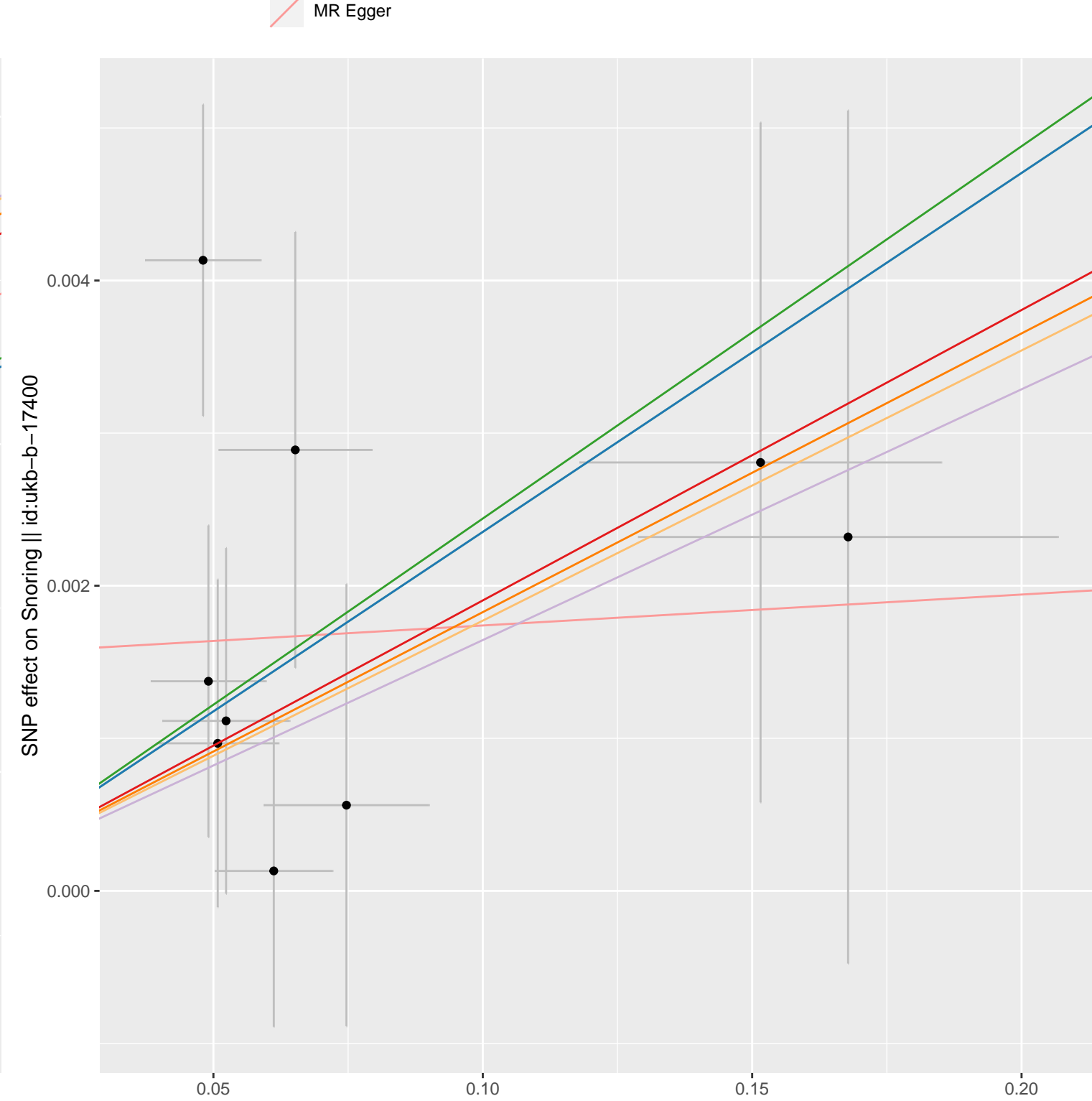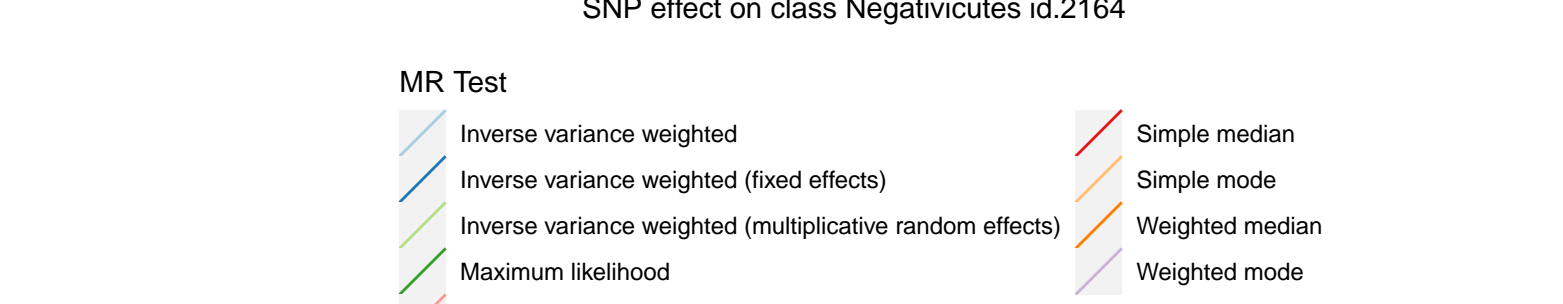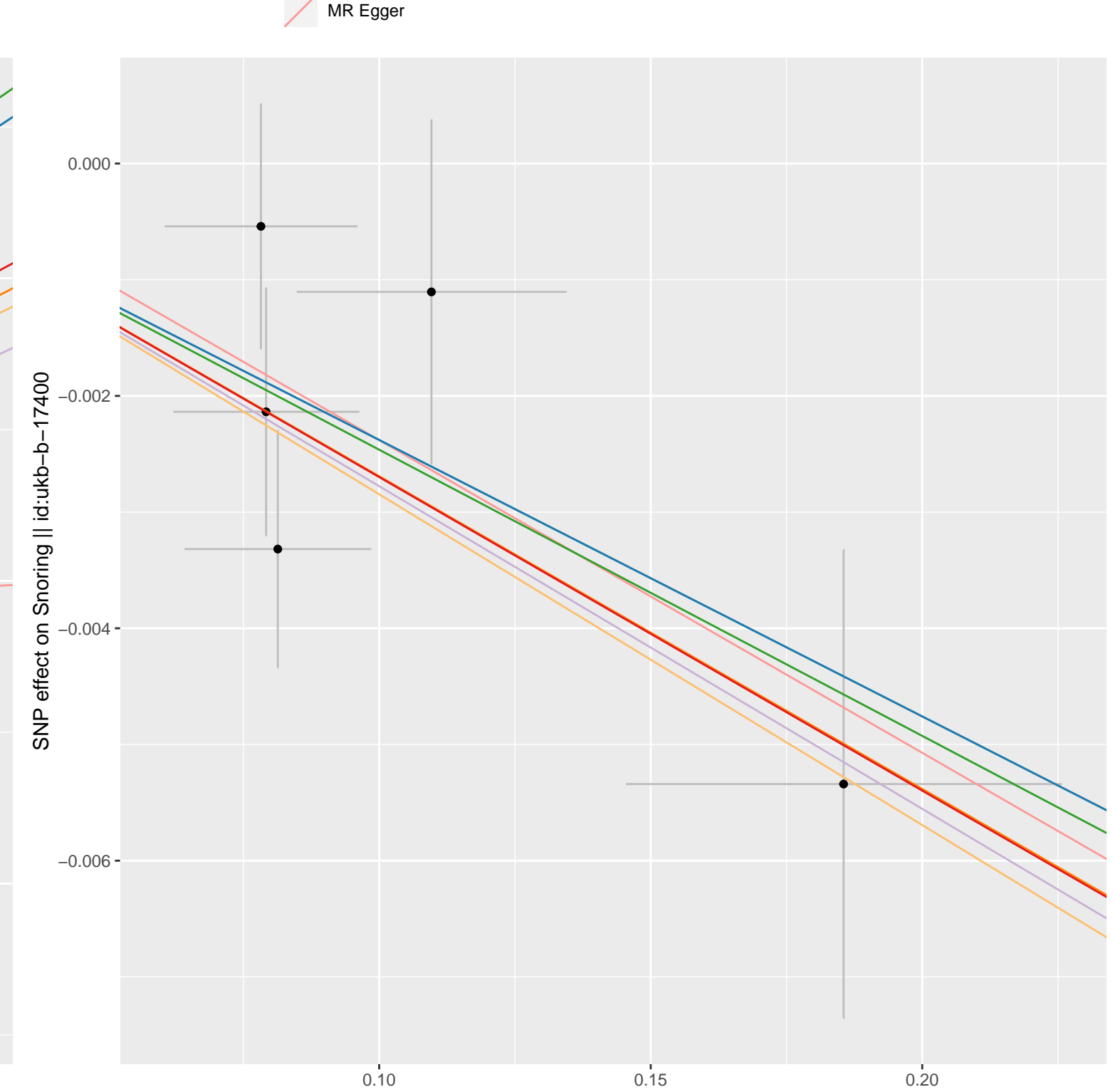

Supplement: Supplementary file 1 [file clockssleep-05-00037-s001.zip › Figure S2.pdf]
